# Large-Scale CRISPRi and Transcriptomics of Staphylococcus epidermidis Identify Genetic Factors Implicated in Lifestyle Versatility

**DOI:** 10.1128/mbio.02632-22

**Published:** 2022-11-21

**Authors:** Michelle Spoto, Johanna P. Riera Puma, Elizabeth Fleming, Changhui Guan, Yvette Ondouah Nzutchi, Dean Kim, Julia Oh

**Affiliations:** a The Jackson Laboratory for Genomic Medicine, Farmington, Connecticut, USA; b The University of Connecticut Health Center, Farmington, Connecticut, USA; Geisel School of Medicine at Dartmouth

**Keywords:** CRISPR-Cas9, large-scale knockdown, CRISPRi, transcriptomics, *Staphylococcus epidermidis*

## Abstract

Staphylococcus epidermidis is a ubiquitous human commensal skin bacterium that is also one of the most prevalent nosocomial pathogens. The genetic factors underlying this remarkable lifestyle plasticity are incompletely understood, mainly due to the difficulties of genetic manipulation, precluding high-throughput functional profiling of this species. To probe the versatility of S. epidermidis to survive across a diversity of environmental conditions, we developed a large-scale CRISPR interference (CRISPRi) screen complemented by transcriptional profiling (RNA sequencing) across 24 diverse conditions and piloted a droplet-based CRISPRi approach to enhance throughput and sensitivity. We identified putative essential genes, importantly revealing amino acid metabolism as crucial to survival across diverse environments, and demonstrated the importance of trace metal uptake for survival under multiple stress conditions. We identified pathways significantly enriched and repressed across our range of stress and nutrient-limited conditions, demonstrating the considerable plasticity of S. epidermidis in responding to environmental stressors. Additionally, we postulate a mechanism by which nitrogen metabolism is linked to lifestyle versatility in response to hyperosmotic challenges, such as those encountered on human skin. Finally, we examined the survival of S. epidermidis under acid stress and hypothesize a role for cell wall modification as a vital component of the survival response under acidic conditions. Taken together, this study integrates large-scale CRISPRi and transcriptomics data across multiple environments to provide insights into a keystone member of the human skin microbiome. Our results additionally provide a valuable benchmarking analysis for CRISPRi screens and are a rich resource for other staphylococcal researchers.

## INTRODUCTION

Sophisticated and accessible sequencing technologies and computational tools to reconstruct complex metagenomes have greatly expanded the catalog of microbial species associated with a wide variety of different ecosystems. Understanding how these different species contribute to their respective ecosystems is one of microbial ecology’s next great challenges.

For this, experimental investigations at the gene level are vital to studying the behavior of any individual organism. Computational approaches leveraging homology to known sequences in model organisms are useful for predicting gene function, but species and strain-specific differences and erroneous or sparse gene annotations preclude a more extensive reliance on computational predictions. However, gold-standard approaches for characterizing gene function, such as gene knockouts, are laborious to create and phenotype on a scale that would allow a similarly rapid and comprehensive investigation of gene function in nonmodel organisms.

With the advent of large-scale methods for gene fitness investigation, such as transposon mutagenesis sequencing (1) and antisense RNA inhibition (2), high-throughput gene function screens have provided a wealth of fitness data for an increasing diversity of microbes under different growth conditions (3–5). But even with these methods, these high-throughput investigations could still be impractical for nonmodel microbes, especially those intractable to genetic manipulation. For example, restriction-modification systems may prevent the introduction of foreign DNA, or homologous recombination efficiency may be too low to enable direct modification of chromosomal loci. Fortunately, rapid developments in CRISPR-Cas9 technology have provided a new, powerful toolkit for gene modification and the discovery of gene function (6).

In particular, the development of CRISPR interference (CRISPRi) (7, 8) has opened a new avenue for large-scale studies of gene function in prokaryotes that does not rely on direct genetic manipulation (9–13). CRISPRi is a modification of the CRISPR-Cas system that uses a catalytically inactive Cas9 protein (dCas9) for transcriptional repression. Rather than inducing a double-stranded break at a target sequence, which can be lethal in prokaryotes that have limited repair mechanisms, the dCas9 protein, guided by the targeting guide RNA, inhibits transcription of the target sequence via steric hinderance (14). In prokaryotes, CRISPRi has been used for gene essentiality screening (10, 13), to map noncoding RNAs to growth phenotypes (13), and to investigate metabolic functions (15).

Here, we apply CRISPRi to the high-throughput gene fitness screening of a keystone skin commensal bacteria, Staphylococcus epidermidis. We and others have shown that S. epidermidis has key roles in maintaining the integrity and health of the skin microbiome, for example, through modulation of the immune system (16–19), the prevention of colonization by more virulent pathogens (20–22) such as S. aureus, and suppression of virulence (23). However, as a common cause of bloodstream and medical device infections, S. epidermidis is also implicated in skin disease (24, 25) and is a leading nosocomial pathogen (26), prolonging hospital stays and increasing patient morbidity (27, 28). Yet, it is commonly assumed that these S. epidermidis infections are seeded from patients’ own skin, where it is ubiquitous (29, 30) and surprisingly diverse within an individual, with unique skin site specification and functional niche adaptation (23). We speculate that the genetic diversity of S. epidermidis enables this lifestyle versatility as a ubiquitous skin colonizer and opportunistic pathogen.

Despite its importance in skin and infectious disease, far less is understood about the growth and survival of S. epidermidis than its more virulent cousin S. aureus. Indeed, nearly 40% of genes lack adequate annotation due to limited genetic manipulation of S. epidermidis to date. Indeed, due to an active restriction-modification system (31, 32) and a limited ability to undergo homologous recombination, classical techniques such as gene knockout or transposon mutagenesis are technically challenging and time-consuming to implement in S. epidermidis (33). As a human health-associated microbe and a difficult-to-study organism, S. epidermidis is an excellent candidate for high-throughput gene fitness screening with CRISPRi.

Here, we generated a high-throughput investigation of gene fitness in S. epidermidis using a large-scale CRISPRi knockdown pool of ~14,000 strains. We screened this pool across 24 diverse stress and nutrient-limiting conditions selected to identify genes that could contribute to the diverse metabolic needs of S. epidermidis for its lifestyle versatility. To further probe gene function, we complemented our fitness analysis with RNA sequencing (RNA-seq) on a subset of 18 of these conditions to investigate the transcriptomic adaptation to these environments. Overall, we identified putative essential genes required for S. epidermidis survival across varied environmental landscapes, with amino acid metabolism, ion uptake, and nitrate/nitrite reduction genes particularly implicated in its lifestyle versatility. We also comment on potential limitations of CRISPRi given inherent species-specific characteristics and pilot droplet-based CRISPRi for increased throughput and sensitivity. This data set represents a large-scale demonstration of CRISPRi in staphylococci and is a resource for characterizing gene fitness and expression across a diversity of environmental conditions for staphylococci.

## RESULTS

### Pooled CRISPRi can identify known essential genes.

**FIG 1:**
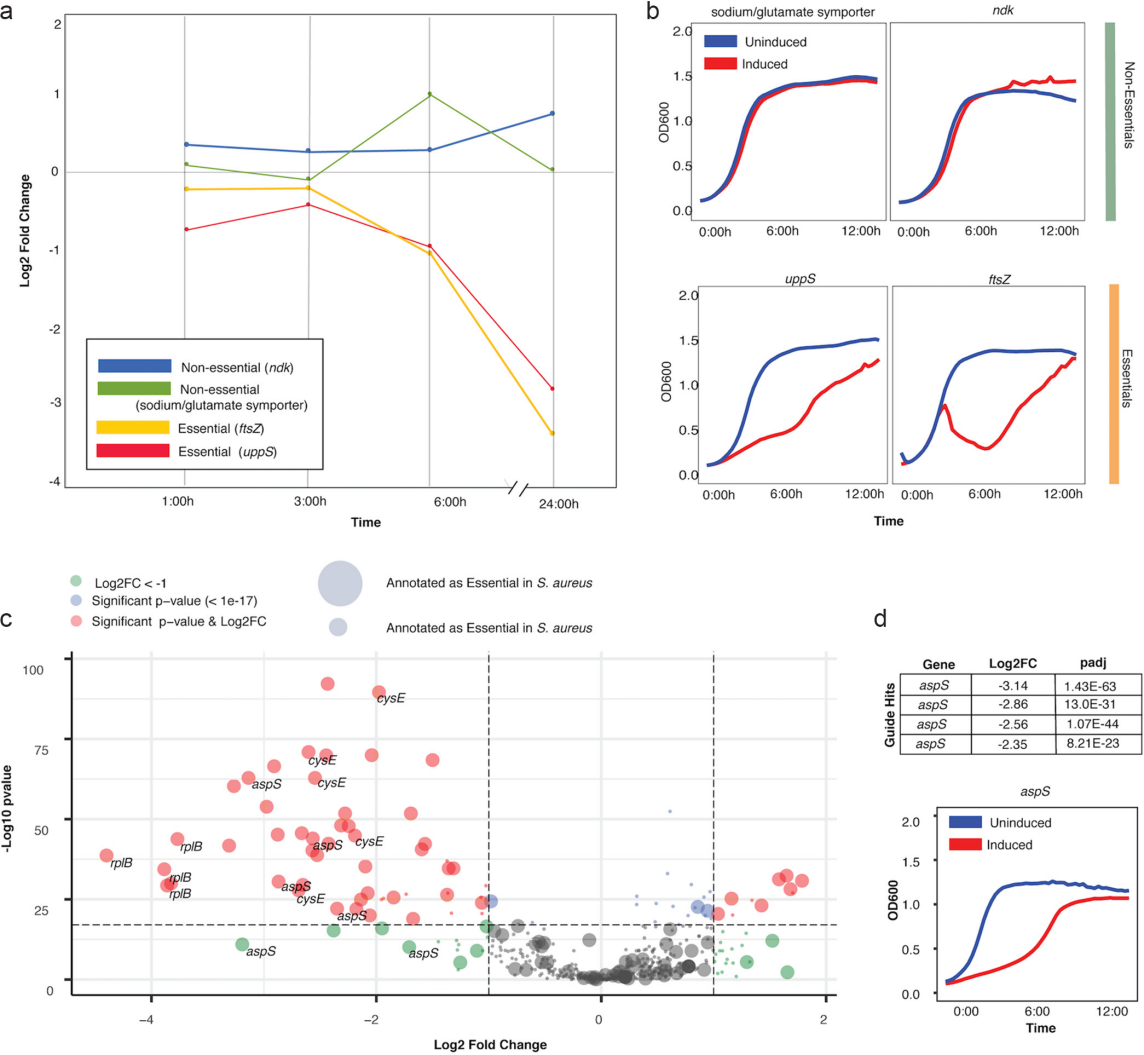
Pooled CRISPRi identifies known essential genes. (a) Line chart depicting log_2_(FC) (final pool/initial pool) of essential and nonessential gene knockdowns across a 24-h growth assay. (b) Individual growth assays across 16 h for each of these four strains depicting growth defects for essential gene knockdowns. (c) Volcano plot of FDR-adjusted *P* value (“padj”) and log_2_(FC) values for the pool of ~500 knockdown strains. Guides with a significant padj value and a log_2_(FC) of <−1 are depicted in red. Guides targeting known essential genes are depicted as large circles; known nonessential genes are depicted as small circles. All guides targeted to a select number of known essential genes (*rplB*, *cysE*, and *aspS*) are shown, depicting that some genes are identified by multiple guides. (d) The log_2_(FC) and padj values for guide hits to *aspS* are depicted alongside an individual growth curve for an *aspS*-knockdown strain. Similar growth curves for *cysE* and *rplB* (and others) can be found in reference 34.

We previously reported our CRISPRi-Cas targeting vector, which contains the necessary CRISPR-Cas machinery for CRISPRi, including dCas9 under the control of an anhydrotetracycline (aTc)-inducible promoter and a combined transactivating CRISPR RNA (tracrRNA)/CRISPR RNA (crRNA) (34). We validated this vector by generating individual knockdown strains of putative essential genes in S. epidermidis and identifying strong growth defects (34). Here, we confirmed that this CRISPRi vector was suitable for identifying essential genes in a pooled screen. First, we generated small “mock” pools with four strains, two strains targeting essential genes and two strains targeting nonessential genes. We induced gene knockdown in these pools, allowed the strains to grow, and determined the relative abundance of each strain at the end of the growth assay. As expected, we saw that strains containing an essential gene knockdown were reduced in abundance in the final pool compared to in the initial pool (Fig. 1a). We used the log_2_(fold change) (log_2_[FC]; defined as the log_2_ of the FC of the normalized read counts of a guide in the final pool/initial pool) to determine the composition of the initial pool versus the final pool after growth. Individual growth assays of these four strains validated detected growth defects (Fig. 1b). Next, we created a larger mock pool of ~500 knockdown strains targeting both putative essential and nonessential genes. Using log_2_(FC) and false discovery rate (FDR)-adjusted *P* values (padj) to call guide “hits,” we found that the majority of these hits were to essential genes (Fig. 1c) and that guides targeting the same gene could yield similar results (Fig. 1d). Taken together, these data confirm the feasibility of our approach.

### Large-scale CRISPRi is effective for identifying gene fitness defects in rich medium (RM).

**(i) Guide design and pool creation.** Using a tiling approach to target the S. epidermidis genome, ~60,000 guides were designed by a customized version of our design script (34). For our proof of principle, we selected S. epidermidis strain Tü3298 (35) for its transformability (36). Versus a gene-based approach for guide design, a tiling approach additionally covers protein-coding sequences on the nontemplate strand, which has been shown to be an effective knockdown strategy for CRISPRi in prokaryotes as well as other genomic elements, for example, unknown promoter regions, rRNA and tRNA sequences, repeat regions, and others (13). Guides were cloned into a previously validated shuttle vector (34). Following microarray-based guide synthesis, amplification, cloning in Escherichia coli, and, finally, phagemid transformation into S. epidermidis, a loss of guides at each step resulted in a final knockdown library containing 14,341 unique guides targeting 1,608 genes (~67% of Tü3298 predicted protein-coding sequences). The number of guides per gene ranged from 1 to 31 depending on gene length and GC content (median of 2 guides per gene). GC content of guides ranged from 35 to 85% (median 42%). The most pronounced limitations to guide design and final pool composition were the low GC content of the S. epidermidis genome (33%; we set a threshold of at least 35% GC content for our guides, in line with previously published reports) and the multistep transformation in S. epidermidis, with a bottleneck at the intermediate S. aureus transformation stage. Full details and additional results on guide design are in Text S1 in the supplemental material. We note that improvements to transformation efficiency are a crucial step in the generalizability of large-scale CRISPR-Cas screens to nonmodel microbes.

10.1128/mbio.02632-22.1 TEXT S1 Guide features and scramble guides. Additional information describing the behavior of scramble guides and data normalization. Download Text S1, PDF file, 0.1 MB. Copyright © 2022 Spoto et al. 2022 Spoto et al. https://creativecommons.org/licenses/by/4.0/ This content is distributed under the terms of the Creative Commons Attribution 4.0 International license.

**FIG 2:**
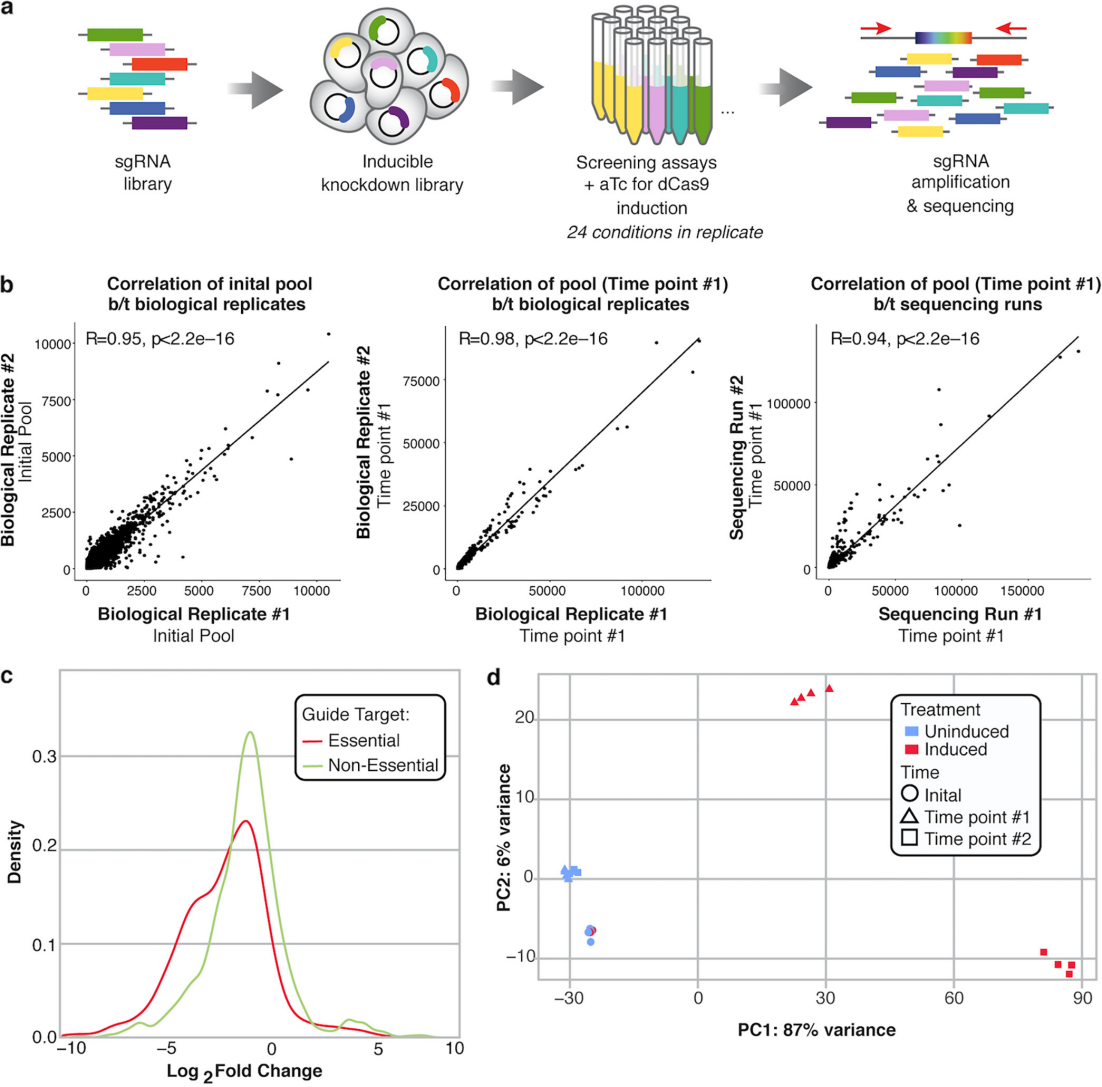
The genome-wide CRISPRi library demonstrates inducible knockdown in rich medium (RM). (a) Overview of library creation and testing. Briefly, guides were designed to all available NGG PAM sites in the S. epidermidis Tü3298 genome. Guides were synthesized on microarray, ligated into a custom anhydrous tetracycline (aTC)-inducible pidCas9 shuttle vector, and transformed into S. epidermidis via sequential transformations into E. coli, S. aureus, and a final phagemid transfer step. The library was screened using 0.1 μM aTC for inducible knockdown across 25 conditions in at least triplicate and sequenced on Illumina HiSeq and NovaSeq platforms. (b) Technical and biological replicates of normalized read counts of the initial knockdown pool (left), after 6 h of induced growth (center), and between sequencing runs (right) are highly correlated (Pearson correlation, top left); b/t, between. (c) Density distribution of the log_2_(fold change) (log_2_[FC]) of guides targeting putative essential (defined by homology to known S. aureus essential genes) and nonessential genes after screening in rich medium, where log_2_(FC) is equal to relative abundance of a guide in the final pool/initial pool (calculated using DeSeq2). (d) PCoA plot of normalized read counts of the initial pool, induced, and uninduced samples after growth to early- (time point 1) and mid-exponential phase (time point 2). Uninduced samples grown to exponential phase (blue triangles and squares) cluster together near the initial pool (blue circles), which indicates even growth of the control pool. Induced samples grown to exponential phase (red triangles and red squares) cluster together by time point away from the initial pools and uninduced pools, which indicates the impact of induction on the final composition of the pool.

**(ii) CRISPRi screening in rich medium (RM) is reproducible and can discriminate essential versus nonessential genes.** Next, we characterized the reproducibility of our CRISPRi knockdown library and the ability to identify strains with growth defects using rich medium (tryptic soy broth [TSB]) as our baseline. We induced gene knockdown by dCas9, allowed the library to grow in rich medium, and used the log_2_(FC) of normalized read counts at endpoint to identify which knockdown strains were significantly depleted in the induced pool compared to an uninduced control pool after growth (Fig. 2a). First, to examine reproducibility, we found that the composition of the knockdown library was highly correlated between biological replicates and between sequencing runs (Fig. 2b; Pearson correlation of normalized read counts; *R* = 0.95, *R* = 0.98, and *R* = 0.95, respectively.) As anticipated, the distribution of the log_2_(FC) (uninduced, no knockdown pool/induced, knockdown pool) for predicted essential genes demonstrated a negative shift compared to predicted nonessential genes (Fig. 2c). Additionally, induction of gene knockdown affected the overall composition of the library. Principle-coordinate analysis (PCoA) of normalized read counts (Fig. 2d) differentiated no-knockdown (uninduced) samples, while knockdown (induced) samples clustered together by time point (Fig. 2d), indicating increasing knockdown phenotype over longer growth periods. These results confirm the feasibility of using our large-scale knockdown pool for robust identification of genes that affect growth *in vitro*.

### CRISPRi screening identifies putative essential and stress response genes across multiple conditions.

After initial characterizations in rich medium, we extended our gene phenotyping to 24 *in vitro* conditions (Table 1) that were selected with the goal of providing insights into the basic physiological response of S. epidermidis to stress and growth in different environments. We hypothesized that genes required for S. epidermidis survival across a range of environmental stressors would differ from those identified only in rich medium. First, we sought to identify genes essential for growth irrespective of environmental condition and then examined genes vital for growth under multiple stress conditions.

**TABLE 1:** Cultivation conditions

Abbreviation^*a*^^,^^*b*^	Base medium^*a*^^,^^*b*^	Condition^*a*^^,^^*b*^
RM^*c*^	TSB	NA
DM^*c*^	Defined medium	NA
RM-acidic^*c*^	TSB	HCl to pH 4.8
DM-acidic	Defined medium	HCl to pH 4.8
RM-alkaline^*c*^	TSB	NaOH to pH 9.0
RM-anaerobic^*c*^	TSB	Anaerobic
DM-anaerobic^*c*^	Defined medium	Anaerobic
RM-glycerol^*c*^	TSB	20% wt/vol glycerol (a_w_ = ~0.95)
RM-salt	TSB	7.5% wt/vol NaCl (a_w_ = ~0.97)
RM-sucrose^*c*^	TSB	40% wt/vol sucrose (a_w_ = ~0.95)
RM-urea^*c*^	TSB	125 μg/mL ciprofloxacin
RM-ciprofloxacin	TSB	4.5% wt/vol urea (a_w_ = ~0.97)
RM-mupirocin^*c*^	TSB	125 ng/mL mupirocin
RM-vancomycin^*c*^	TSB	1.75 μg/mL vancomycin
RM-HOCl^−^^*c*^	TSB	0.015% vol/vol HOCl^−^
RM-H_2_O_2_^*c*^	TSB	0.0055% vol/vol H_2_O_2_
RM-42°C	TSB	42°C
DM-30°C	Defined medium	30°C
DM-low iron^*c*^	Defined medium	1 μM FeCl_3_
DM-high iron	Defined medium	100 μM FeCl_3_
MM-high AA, high glucose^*c*^	M9 minimal medium	1% wt/vol Casamino Acids 1% wt/vol glucose
MM-high AA, low glucose^*c*^	M9 minimal medium	1% wt/vol Casamino Acids 0% wt/vol glucose
MM-low AA, high glucose^*c*^	M9 minimal medium	0.1% wt/vol Casamino Acids 1% wt/vol glucose
MM-low AA, low glucose^*c*^	M9 minimal medium	0.1% wt/vol Casamino Acids 0% wt/vol glucose

a Gene fitness screening was conducted under 24 diverse conditions that were chosen to reflect stressors potentially encountered in colonization and infection.

b DM, defined medium; RM, rich medium; MM, minimal medium; NA, not applicable; AA, amino acid; TSB, tryptic soy broth; a_w_, water activity.

c Condition also used for transcriptional profiling.

**FIG 3:**
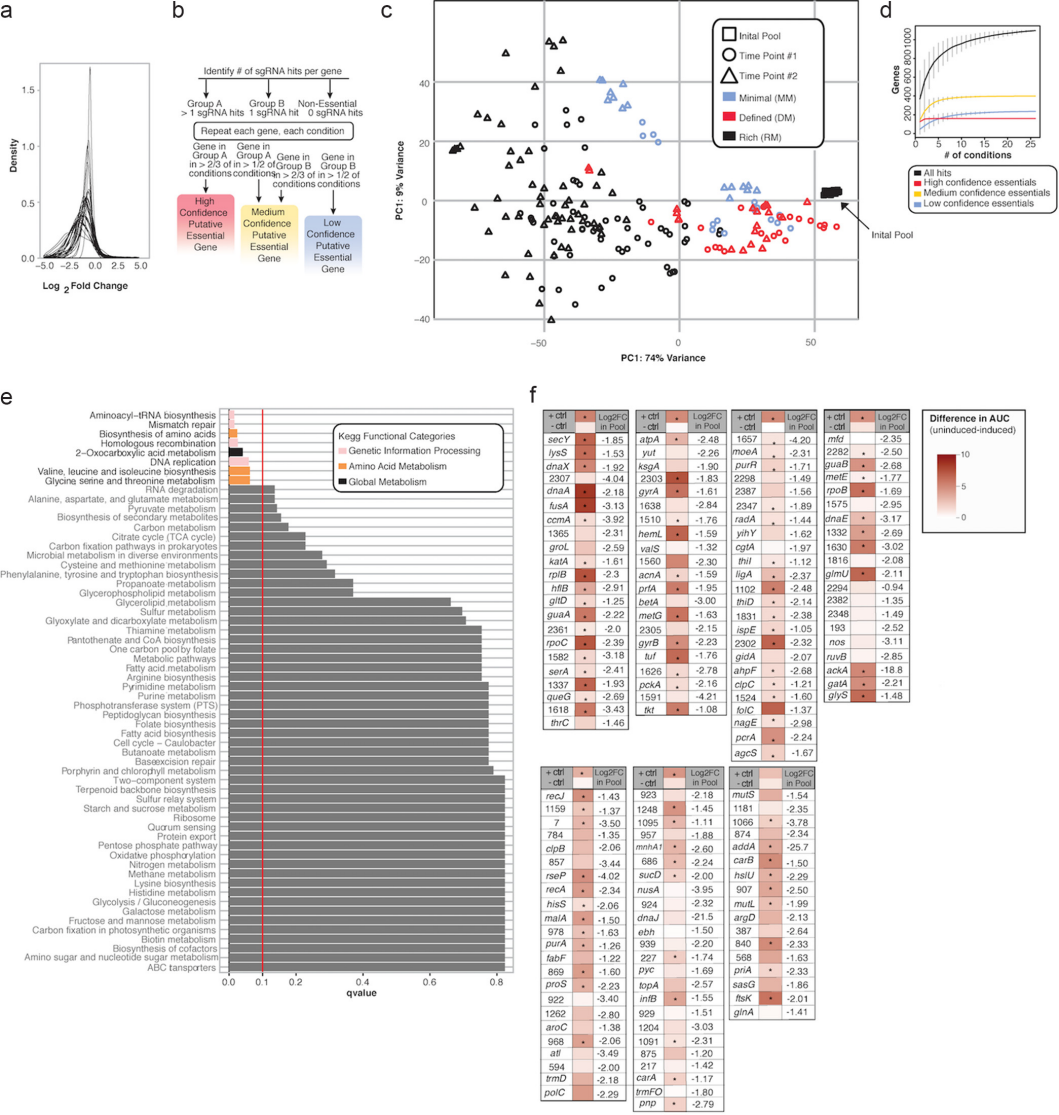
Multicondition CRISPRi identifies putative essential genes. (a) Various log_2_(FC) distributions across 24 conditions. (b) Workflow of putative essential gene identification across conditions. Identified genes were binned into high-, medium-, or low-confidence categories based on quality of supporting data as described. (c) PCoA plot of normalized read counts demonstrating segregation of samples by medium type and time point after aTC induction (the initial pool is also plotted, clustered as rightmost overlapping black squares). (d) Gene accumulation curves for high-, medium-, and low-confidence putative essential or nonessential genes across increasing numbers of conditions. (e) Enriched KEGG pathways within the high- and medium-confidence essential gene list. Significant pathways (as determined by default GAGE analysis [parametric *t* test and multiple testing correction to control FDR]; FDR < 0.10) are colored by KEGG category; gray pathways indicate nonsignificance. The vertical red line indicates our significance threshold of an FDR *P* value of 0.10. (f) Validation of high-confidence hits. The difference in area under growth curve (AUC) was calculated as uninduced AUC (i.e., no knockdown) – induced (i.e., knockdown) AUC and is depicted in red. Statistical significance was determined by Student’s *t* test for AUC uninduced versus induced; *n* = 3; *, *P* < 0.05; +ctrl is known essential gene Tü3298_1803 (ribosomal protein L2), and −ctrl is known nonessential gene Tü3298_1121 (nucleoside diphosphate kinase). The *P* value for +ctrl in the last batch is 0.055. The gene name is provided if annotated, otherwise the numbers represent Tü3298 gene IDs.

**(i) Essential gene identification.** Due to the various growth rate of S. epidermidis across conditions and the level of growth that each of these conditions supports, we observed various log_2_(FC) distributions across conditions (Fig. 3a). Thus, we decided against a strict log_2_(FC) cutoff for gene hits, which would have the effect of over- or underidentifying potential “hits” depending on the distribution. Instead, we called hits individually for each condition and then leveraged these data across all conditions to determine a set of “high-,” “medium-,” and “low-confidence” putative essential genes (Fig. 3b), identifying 160, 442, and 304, respectively (see Table S1 for the high-confidence essential gene list; medium- and low-confidence gene lists can be obtained as noted in in the Data availability). In addition, to circumvent relying on limited genome annotation and unvalidated operon prediction in S. epidermidis, we opted to call hits based on individual genes irrespective of operon organization. As polar effects were estimated to result in a 15% false-positive rate in the discovery of essential genes in a CRISPRi screen in E. coli (12), we anticipate that these effects do not represent a major limitation to our study.

10.1128/mbio.02632-22.2 TABLE S1 Guides designed by a customized version of our GuideFinder script for large-scale CRISPRi targeting. Download Table S1, XLSX file, 0.01 MB. Copyright © 2022 Spoto et al. 2022 Spoto et al. https://creativecommons.org/licenses/by/4.0/ This content is distributed under the terms of the Creative Commons Attribution 4.0 International license.

We then examined consistency between conditions as a quality control check and to identify possible technical variation between our diverse conditions. First, we grouped our conditions by background medium type as rich (“TSB”), defined (“defined media” [DM]), or minimal (“M9 minimal media” [M9 MM]) (Table 1) and generated a PCoA plot of the normalized read counts. As expected, the initial pool samples clustered together, and there was a slight differentiation between the background medium types (Fig. 3c). Gene accumulation curves were generated for high, medium, and low confidence putative essential genes across an increasing number of tested conditions.

Next, we sought to determine the consistency of our screening results across conditions. With the exception of condition-specific essential genes for each environment, we expected to see that a gene behaves similarly (e.g., essential or nonessential) across conditions. To determine this, we generated heatmaps of the conditions grouped by medium type (median log_2_[FC] of all guides for each gene versus condition). We observed that the median log_2_(FC) of each gene was largely consistent between conditions, suggesting little technical variability between screen conditions (Fig. S2a to c).

10.1128/mbio.02632-22.7 FIG S1 Scramble sgRNAs behave distinctly within the CRISPRi screen. (a) Distribution of the log_2_(FC) values for guides targeting putative essential and nonessential genes and scramble sgRNAs demonstrates aberrant behavior of strains containing scramble sgRNAs. (b) Data demonstrating the same distributions when estimation of size factors is based on scramble sgRNA. Consequently, we deem estimation of size factors based on scrambled sgRNAs inappropriate. Download FIG S1, TIF file, 0.03 MB. Copyright © 2022 Spoto et al. 2022 Spoto et al. https://creativecommons.org/licenses/by/4.0/ This content is distributed under the terms of the Creative Commons Attribution 4.0 International license.

10.1128/mbio.02632-22.8 FIG S2 Median log_2_(FC) values for genes is generally consistent between conditions of a medium type. (a to c) Heatmaps of the median log_2_(FC) of genes across conditions grouped by medium type (minimal [a], defined [b], and rich [c]). Hierarchical clustering was generated based on Euclidean distances. Download FIG S2, TIF file, 0.07 MB. Copyright © 2022 Spoto et al. 2022 Spoto et al. https://creativecommons.org/licenses/by/4.0/ This content is distributed under the terms of the Creative Commons Attribution 4.0 International license.

Next, we generated gene accumulation curves for our putative essential genes (high, medium, and low confidence) across our 24 conditions. We then sought to examine the broad functional classifications of these putative essential genes. We performed a KEGG enrichment analysis on the medium- and high-confidence gene lists compared to the Tü3298 background and identified enrichment in pathways involved in genetic information processing, amino acid metabolism, and global metabolism, including aminoacyl-tRNA synthesis, mismatch repair, and homologous recombination (Fig. 3e). Generally, these results were consistent with the established literature regarding gene essentiality in staphylococci (37, 38) and model microorganisms, which have identified DNA and RNA processing and protein synthesis among key essential functions (39). While previous reports in S. aureus demonstrate that genes involved in amino acid metabolism are not consistently identified as essential in rich medium (37, 38), amino acid metabolism has been hypothesized to play a role in the functional adaptation of S. aureus to diverse nutrient environments (40). Our results suggest that amino acid biosynthesis and metabolism are core mechanisms underlying the success of S. epidermidis across a range of diverse nutrient and stress-related conditions.

Finally, we gauged the ability of the pooled assay to reliably identify genes whose knockdown affects growth. We created 151 individual knockdowns of the list of high-confidence essential genes and tested their growth rate in rich medium (Fig. 3f). Despite differences afforded by competitive growth in the pooled assay, taken together with sequencing-based quantitation, which can identify subtle growth defects, we demonstrated that the majority of these high-confidence hits also demonstrated a growth defect when grown in single culture versus a no-knockdown strain.

**(ii) Multistress response gene identification.** We then asked whether we could use the CRISPRi screen data to identify stress response genes, that is, those with a fitness defect under multiple stress conditions, which may represent genes responsible for adaptation to stress or other environmental signals. By screening across multiple stressors, we could again identify high-confidence hits, leveraging the utility of the pooled CRISPRi approach. To control for the effect of background medium type (e.g., rich versus minimal) on gene fitness, we restricted our study of multistress response genes to stress screens conducted in rich medium (TSB). These conditions included multiple antibiotic stress conditions, multiple hyperosmotic stress conditions, oxidative stress conditions, and more (Table 1). We designated a gene as a multistress response gene if it was identified in at least ~50% (6/13) of these rich-medium stress conditions but was not identified as a hit in plain rich medium or as a putative essential gene (high or medium confidence). We identified 25 such genes (Table 2), several of which have known functions in stress response or DNA or protein damage repair (e.g., *ssrB*, *msrA*, *uvrC*, and *dinG*) (41–47). Of particular interest are previously unidentified hits related to inorganic ion transport and metabolism. We identified three genes in this category (*ptsA*, *cntL*, and *cntA*), of which *cntL* and *cntA* are involved in staphylopine biosynthesis and staphylopine-dependent metal transport, respectively. *ptsA* is involved in phosphate and nickel transport, while staphylopine is a broad-spectrum metallophore (48). Trace metals, including nickel, are vital cofactors for enzymatic catalytic activity, and we postulate that demand for these cofactors is increased under cellular stress. We note that of 25 identified genes, 6 have no or poor functional annotation. Given their potential role in response to multiple stressors, these genes are strong candidates for future follow-up investigations. Finally, our analysis also identified *nusB*, a gene annotated as essential in S. aureus. This gene may have been missed by our essential gene screening or represents a true biological difference in gene fitness between the species.

**TABLE 2:** CRISPRi identifies multistress response genes in rich medium

Tü3298 ID^*a*^	Name	Symbol	Function
Tü3298_95	rRNA methyltransferase family protein	*cntL*	Staphylopine biosynthesis
Tü3298_97	Nickel ABC transporter, nickel/metallophore periplasmic binding protein	*cntA*	Trace metal import
Tü3298_1030	Phosphate ABC transporter, permease protein PstA	*pstA*	Transport; nickel and phosphate import
Tü3298_149	Transporter, betaine/carnitine/choline transporter family protein	—^*b*^	Transport; osmoprotectant
Tü3298_1053	Cobalamin biosynthesis CobT VWA domain protein	—^*b*^	Inorganic ion transport and metabolism
Tü3298_1077	Peptide-methionine (*S*)-*S*-oxide reductase	*msrA*	Protein damage repair
Tü3298_1108	Exonuclease, DNA polymerase III, epsilon subunit family domain protein	—^*b*^	DNA damage response
Tü3298_795	Excinuclease ABC subunit C	*uvrC*	DNA damage response
Tü3298_1138	Sensor protein SrrB	*srrB*	Signal transduction and stress response
Tü3298_2206	PTS^*c*^ system mannose-specific EIIBCA component	*manP*	Carbohydrate metabolism
Tü3298_370	1-Phosphofructokinase	*pfkB*	Carbohydrate metabolism
Tü3298_797	Succinate dehydrogenase/fumarate reductase, flavoprotein subunit	*sdhA*	Energy production and conservation; TCA cycle
Tü3298_1955	Nitrate reductase, alpha subunit	—^*b*^	Energy production and conservation; nitrate reductase
Tü3298_1106	Asparagine-tRNA ligase	*asnS*	Translation
Tü3298_1465	RNA pseudouridylate synthase family protein	—^*b*^	Translation
Tü3298_1054	Sigma-54 interaction domain protein	—^*b*^	Nitric oxide reductase
Tü3298_1133	Elastin-binding protein EbpS	*ebpS*	Adhesion protein
Tü3298_698	2-Succinyl-5-enolpyruvyl-6-hydroxy-3-cyclohexene-1-carboxylic-acid synthase	*menD*	Coenzyme metabolism
Tü3298_1167	Transcription antitermination factor NusB	*nusB* ^*d*^	Transcription
Tü3298_108	Conserved hypothetical protein	—^*b*^	Unannotated
Tü3298_116	C4-Dicarboxylate anaerobic carrier family protein	—^*b*^	Unannotated
Tü3298_2208	yhgE/Pip C-terminal domain protein	—^*b*^	Unannotated
Tü3298_181	kxYKxGKxW signal peptide domain protein	—^*b*^	Unannotated
Tü3298_778	Conserved hypothetical protein	—^*b*^	Unannotated
Tü3298_2157	Putative membrane protein	—^*b*^	Unannotated

a Gene was designated a multistress response gene if it was identified as a hit by multiple guides in at least 50% of the rich medium stress conditions.

b — indicates no data.

c PTS, phosphotransferase system.

d Annotated as essential in S. aureus.

Taken together, we demonstrate how high-throughput CRISPRi screens across diverse growth conditions can identify essential gene and multistress response gene candidates. With stringent thresholds for gene hit identification (at least 2 independent guide hits per gene and analyses conducted across multiple conditions in at least biological triplicate), these results likely represent robust phenotypes. Our individual confirmation of high-confidence essential genes further supports the validity of such screens.

### CRISPRi screening identifies condition-specific gene hits.

We then sought to identify specific genes vital to growth under each condition, which could provide insights into the lifestyle versatility of S. epidermidis. By identifying hits specific to each environment, we could also construct an accessible resource of gene fitness data for S. epidermidis. Here, we considered condition-specific genes as hits identified under a particular condition but not considered a high- or medium-confidence putative essential gene (i.e., those identified across all conditions; data are available as described in Data availability). We note that an inherent limitation of our approach is that our library does not cover every protein-coding sequence, and, as such, the absence of a gene as a hit should not be taken as evidence that the gene is not conditionally essential. Instead, this condition-specific essentiality screen provides high-quality hits that constitute a starting point for investigating genes of interest.

**(i) CRISPRi screening suggests a role for urease and cell wall function in response to salt stress.** As an example, we asked whether we could identify genes required for survival under high salt stress, a stress condition that S. epidermidis may face on the surface of human skin. After performing the pooled fitness assay in 7.5% sodium chloride, we identified several condition-specific hits previously identified in the literature as vital under high salt stress, including a cation transporter, genes of the *dlt* operon (implicated in cell wall biosynthesis), and a betaine/carnitine/choline transporter (49). We also found several previously unidentified hits, including two urease accessory coenzymes involved in nickel binding (*UreG* and *UreE*), a transcriptional regulator annotated only as involved in “cell envelope-related function” (Tü3298_708), and a teichoic acid d-alanine hydrolase (*fmtA*). Although the exact function of *fmtA* is unknown, it is implicated in cell wall structure and methicillin resistance in S. aureus (50). Given the role of cell wall structure in response to hyperosmotic stress, we speculate that this is the basis for the essentiality of *fmtA* and the putative transcriptional regulator gene under high salt stress conditions. The role of the accessory urease chaperones in high salt stress is a curious find and, taken together with other high-quality hits (*secA2*, *purH*, and *purM*), is of interest for follow-up investigation into the role of salt tolerance in S. epidermidis.

### RNA-seq identifies pathways involved in S. epidermidis stress response and growth under diverse nutrient conditions.

**FIG 4:**
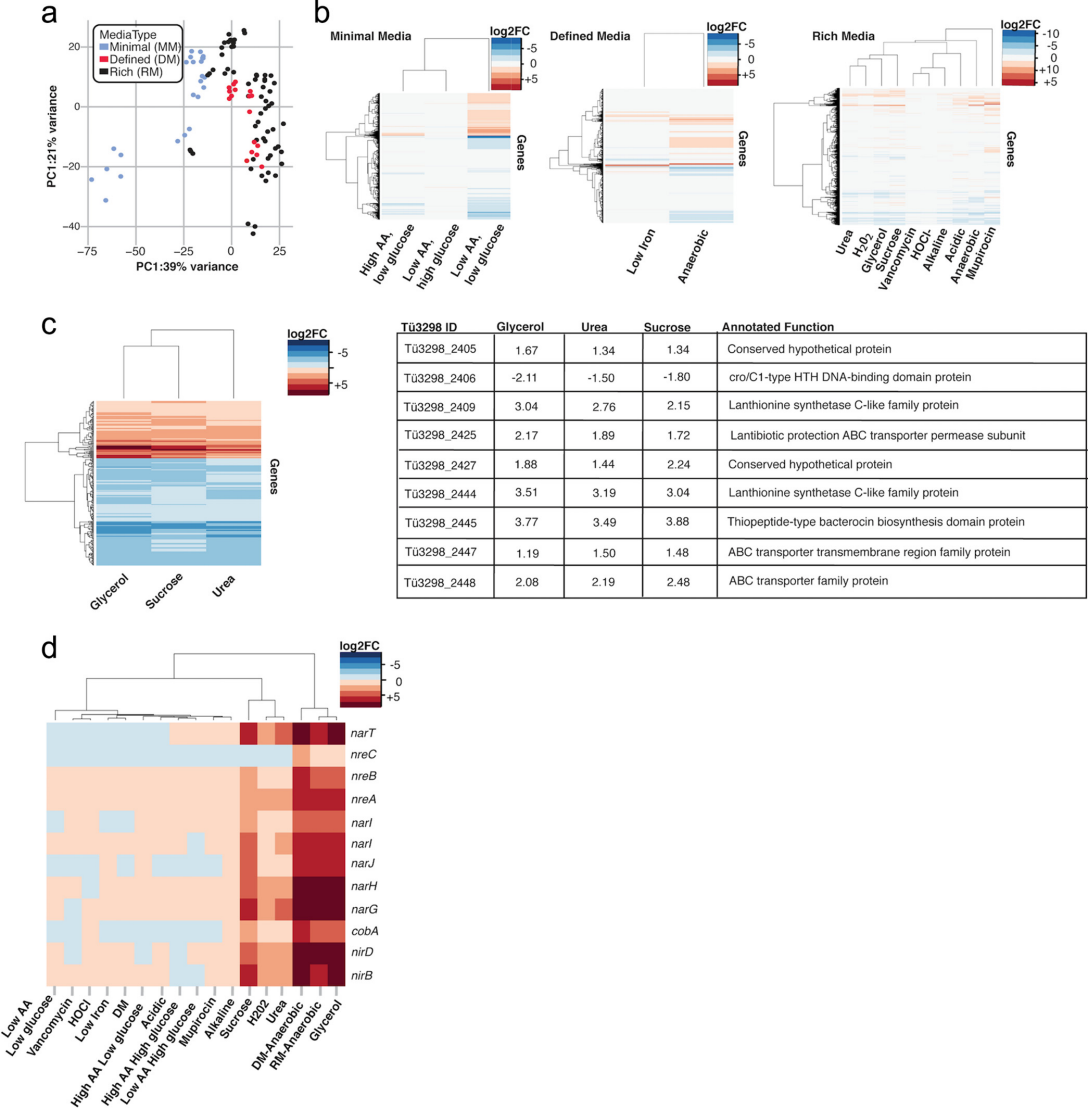
Matched RNA-seq analysis of S. epidermidis grown under CRISPRi assay conditions identifies stress-specific responses. (a) PCoA plot of normalized read counts depicting clustering of RNA-seq samples (*n* = 3 per condition) by medium type. (b) Heatmaps of log_2_(FC) values for each condition grouped by medium type. The reference for RM conditions is plain RM (TSB), for DM conditions is plain DM, and for MM conditions is M9 MM supplemented with 1.0% Casamino Acids (CAA) and 1.0% glucose (high-amino acid [high-AA], high-glucose condition). (c) Left, heatmap of genes upregulated (log_2_[FC] > 1) or downregulated (log_2_[FC] < −1) under all of the osmotic stress conditions. A table of genes involved in lantibiotic synthesis and export differentially expressed under all three osmotic stress conditions is shown on the right. (d) Heatmap of genes involved in nitrate/nitrite reduction across conditions compared to RM. Hierarchical clustering for b, c, and d was based on Euclidean distances.

**(i) Overview of transcriptomics analysis across conditions.** While fitness investigations identify genes necessary for growth under stress conditions or under nutrient limitation, RNA-sequencing studies define the global transcriptional plasticity of an organism. Indeed, it is well established that differential expression of a given gene is a poor indicator of gene fitness under stress (i.e., many differentially expressed genes [DEGs] are not essential), and, thus, transcriptomic analysis provides a complementary approach to studying lifestyle versatility in S. epidermidis. A subset of 18 conditions from the CRISPRi screening assays, selected to represent a diversity of stressors across minimal, defined, and rich background media, was selected for RNA-seq to examine pathways involved in the response of S. epidermidis to growth under diverse conditions. To broadly examine S. epidermidis adaptation across conditions, we first grouped conditions by background medium type. We anticipated that nutrient availability would have the strongest influence on transcription, which was confirmed by PCoA analysis (Fig. 4a). Analysis of log_2_(FC) values for each condition further demonstrated that broad transcriptional patterns were driven by the underlying background medium type, which was the basis of our medium-type-specific analysis below (Fig. 4b).

**(ii) Global transcriptomic analysis identifies genes enriched under hyperosmotic stress conditions.** One advantage to performing transcriptomic analysis across a variety of stress conditions is the ability to identify overlapping responses between conditions within a stressor type (e.g., osmotic stress, antibiotic stress, and oxygen limitation) to further inform cellular response. As an example, we sought to identify a subset of genes globally responsive to hyperosmotic stress. We identified 209 genes significantly upregulated under all three hyperosmotic stress conditions (sucrose, glycerol, and urea) with a log_2_(FC) cutoff of 0.5 (Table S2) and 81 genes with a more stringent log_2_(FC) of 1.0 (Table S3; Fig. 4c, left). KEGG pathway analysis on the most highly enriched genes (log_2_[FC] ≤ 1) identified the following categories as significantly enriched (FDR ≤ 0.1) under osmotic stress: nitrogen metabolism, two-component systems, butanoate metabolism, microbial metabolism in diverse environments, glycolysis/gluconeogenesis, purine metabolism, and propanoate metabolism. Consistent with previous reports, we identified a choline-glycine betaine transporter (Tü3298_149) and genes involved in iron uptake (*feoA* and *feoB*) (51–53). Interestingly, multiple genes involved in bacteriocin synthesis were enriched (e.g., genes highlighted in Fig. 4c, right). Specifically, gene clusters involved in epidermin synthesis and export were enriched across all three osmotic stress conditions. Epidermin is a pore-forming antimicrobial peptide effective against a range of Gram-positive bacteria, including S. aureus. While the role of bacteriocin synthesis and export during osmotic stress is not readily apparent, one possibility is that it may be a generalized mechanism to improve competition with other members of the skin microbiome during periods of stress.

10.1128/mbio.02632-22.3 TABLE S2 Genes enriched in all three osmolarity conditions (log_2_[FC] cutoff of ≥0.5); log_2_(FC) values, padj values, and gene annotations are included in this file. Download Table S2, XLSX file, 0.04 MB. Copyright © 2022 Spoto et al. 2022 Spoto et al. https://creativecommons.org/licenses/by/4.0/ This content is distributed under the terms of the Creative Commons Attribution 4.0 International license.

10.1128/mbio.02632-22.4 TABLE S3 Genes enriched in all three osmolarity conditions (log_2_[FC] cutoff of ≥1.0); log_2_(FC) values, padj values, and gene annotations are included in this file. Download Table S3, XLSX file, 0.02 MB. Copyright © 2022 Spoto et al. 2022 Spoto et al. https://creativecommons.org/licenses/by/4.0/ This content is distributed under the terms of the Creative Commons Attribution 4.0 International license.

**(iii) Global transcriptomic analysis identifies nitrogen reduction as enriched during anaerobic, hyperosmotic, and hydrogen peroxide stress.** We were also surprised to find genes involved in nitrogen metabolism as some of the most highly enriched under all three hyperosmotic stress conditions. We saw significant enrichment of genes involved in nitrate/nitrite transport (*narT*), oxygen regulation of nitrate/nitrite reduction (*nreA* and *nreB*), nitrate reduction (*nar* operon), and nitrite reduction (*nir* operon) (Fig. 4d). While a few studies have suggested overexpression of the nitrate or nitrite reductase complexes during osmotic stress, no clear mechanism has been elucidated (54, 55). In a study of uropathogenic E. coli, Withman et al. speculated that by linking hyperosmotic gene expression to anaerobic gene expression (e.g., overexpression of nitrite/nitrate reductases), E. coli may adapt more readily to the hyperosmotic and hypoxic environment of the urethra and bladder lumen. Similarly, S. epidermidis must combat hyperosmotic stress on the skin that results as salt- and urea-rich sweat evaporates from the surface. We speculate that the upregulation of nitrate/nitrite reduction genes in response to hyperosmotic stress provides an avenue for S. epidermidis to use nitrogen from sweat.

Given this striking finding, we then asked whether these genes were similarly enriched under any other stress conditions compared to rich medium. Enrichment of nitrate/nitrite reduction genes is expected under anaerobic conditions, where nitrate is used as an electron acceptor during respiration, and, indeed, these genes are enriched in both anaerobic defined and rich medium. In addition to enrichment under hyperosmotic stress, nitrate/nitrite reduction genes were significantly upregulated in response to hydrogen peroxide stress (Fig. 4d). Under this condition, we also observed upregulation of additional anaerobic metabolic genes, including *pflB* (log_2_[FC] = 4.29) and *nrdG* (log_2_[FC] = 2.12), among others. S. aureus has been shown to upregulate genes involved in anaerobic metabolism in response to hydrogen peroxide-induced oxidative stress, indicating that the microbe undergoes an oxygen-limiting state after exposure (56). Our findings here suggest a similar mechanism for S. epidermidis response to hydrogen peroxide stress.

**FIG 5:**
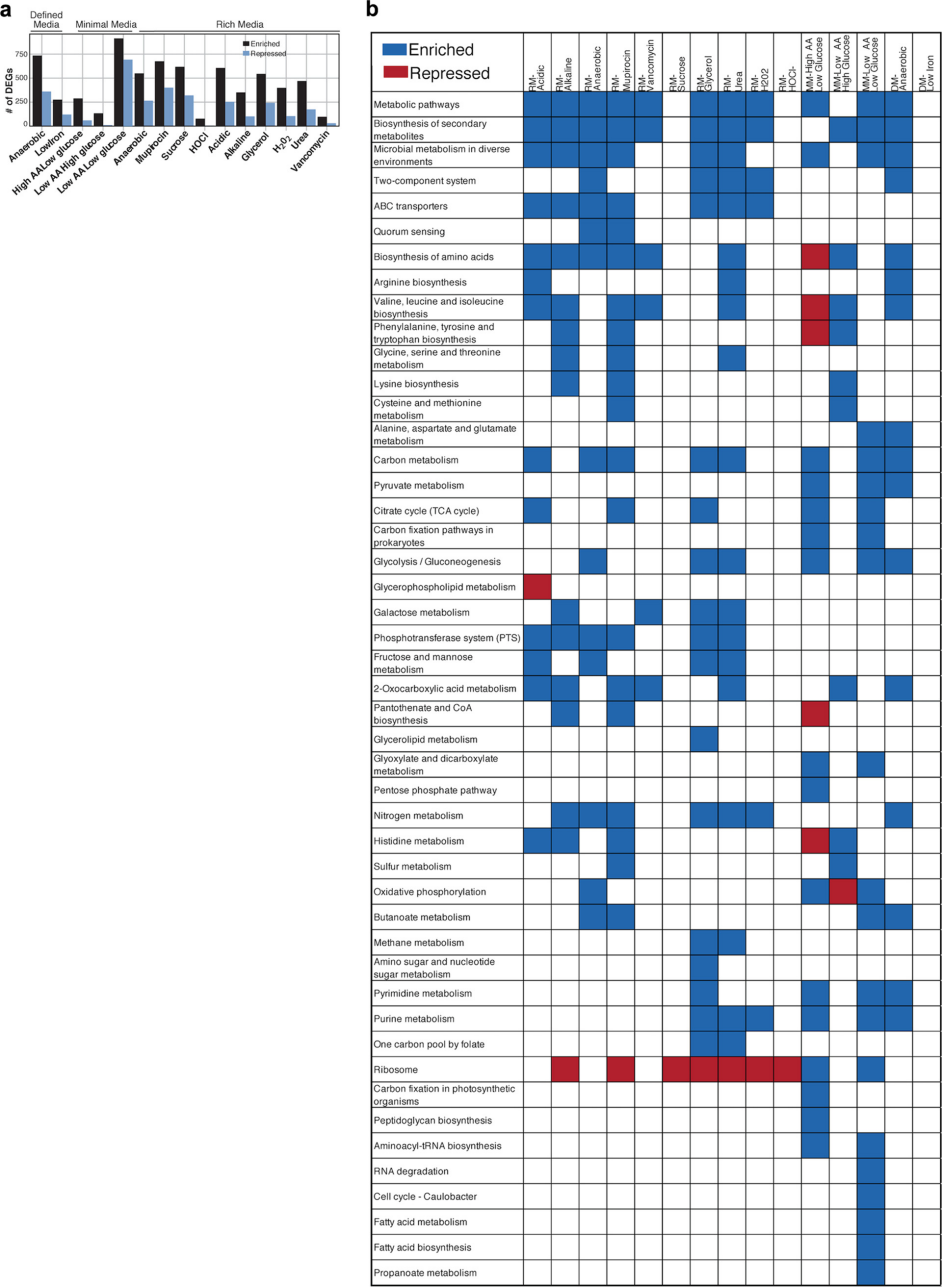
RNA-seq identifies pathways involved in S. epidermidis plasticity across diverse environments. (a) Number of DEGs for each condition grouped by medium type. (b) The top 5 KEGG pathways most significantly enriched and repressed under each condition are grouped by medium type. RM samples are compared to RM baseline medium, DM samples are compared to DM baseline medium, and MM samples are compared to high-amino acid (1.0% Casamino Acids [CAA]), high-glucose (1.0%) minimal medium. The plus sign (+) represents significantly upregulated KEGG pathways at an FDR of ≤0.10; a minus sign (−) represents significantly downregulated pathways at an FDR of ≤0.10. For all KEGG pathways significantly up and down regulated for each condition, see Data Availability section.

**(iv) Condition-specific RNA-seq generates a rich resource for investigating the transcriptomic response of S. epidermidis across a diversity of conditions.** Next, we examined differentially expressed genes (DEGs) within each condition. By analyzing DEGs and corresponding KEGG pathways, we could define the overall transcriptomic response of S. epidermidis under a variety of environmental conditions. We identified DEGs (log_2_[FC] ≥ 0.5 or ≤ −0.5 and FDR ≤ 0.1) under each condition compared to their background medium type (data availability described in Text S1). Generally, stress and nutrient-limited conditions resulted in significantly more upregulated genes/corresponding KEGG pathways than repressed genes (Fig. 5a), likely reflecting a broad transcriptional stress response or large-scale mobilization of genes to increase resource utilization. Additionally, the top five KEGG categories most significantly enriched and repressed in each condition are depicted in Fig. 5b.

As an example, we investigated the transcriptional response to growth in an anaerobic environment. DEGs and KEGG pathways enriched under anaerobic conditions were generally consistent with previous reports. For example, the most highly upregulated genes were involved in nitrate reductase (*nir* and *nar* operons) and oxygen sensing (*nreB* and *nreC*) (57, 58). Genes of the urease operon were also among those most highly upregulated during anaerobic growth, perhaps as a response to an altered pH under the anaerobic condition. Interestingly, three genes of the beta class of phenol-soluble modulins (PSMβ) were highly enriched under the anaerobic condition (log_2_[FC] = 5.91, 5.77, and 5.53), along with the expression of the quorum sensing *agr* locus. Literature regarding the expression of the quorum sensing system in hypoxia is conflicting; in our strain of S. epidermidis at the least, our results provide evidence for upregulation of quorum sensing genes and downstream effectors during oxygen limitation. We speculate that regulation of the quorum sensing system, which controls virulence and other metabolic processes in staphylococci (59–61), is one mechanism by which S. epidermidis adapts to the differential oxygen levels of infection niches.

### CRISPRi screening and RNA-seq cooperatively identify pathways vital for acid resistance in S. epidermidis.

While CRISPRi and transcriptomics each are powerful tools for probing gene function, integrating these data types could provide complementary data on potential gene function. As an example, we performed such an analysis on the acid stress condition, which is relevant to the widely varying pH in human skin (4.1 to 5.8, depending on skin site, individual, and lifestyle factors [62, 63]). While several strategies have been identified in bacteria to accommodate changes in environmental pH, including proton gradients, generation of alkaline products (e.g., ammonium production and amino acid neutralization pathways), alterations to the cell membrane, metabolic changes, and more (reviewed in reference 64), little is known about the acid response system in S. epidermidis. In addition to examining transcriptomic and gene fitness data, we also piloted a droplet-based CRISPRi approach to identify growth defects in genes that may be masked by the effects of growth in batch culture.

**FIG 6:**
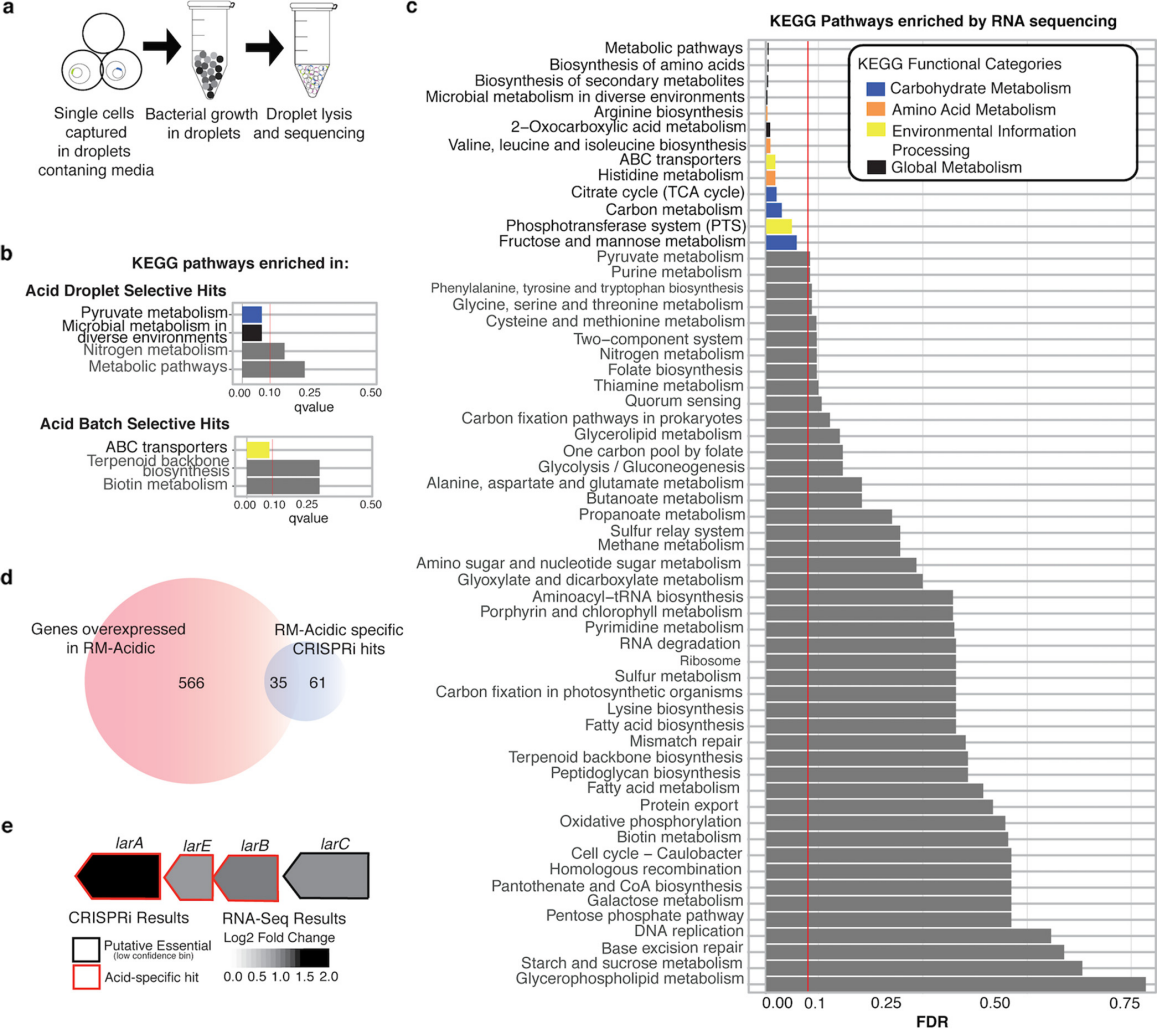
Integrated gene fitness screening and transcriptomics analysis identify cellular functions necessary for survival and adaptability under acid stress. (a) Workflow for droplet-based CRISPRi screening. Briefly, single cells captured in droplets containing RM-acidic medium were incubated to promote growth. After incubation, droplets were lysed, and the lysate was amplified and sequenced as for a batch pool. (b) KEGG pathways enriched in droplet-specific and batch-specific gene lists. Pathways with an FDR of ≤0.25 are shown. Significantly enriched pathways (FDR ≤ 0.1) are colored by KEGG category. (c) KEGG pathways enriched in RM-acidic medium (versus RM) by RNA-seq. Statistically significant results are colored by KEGG category; gray bars represent nonsignificant results. The vertical red line indicates our significance threshold of an FDR of 0.10. (d) Venn diagram depicting overlap of genes overexpressed in acidic medium and genes identified as acid-stress-specific hits by CRISPRi. (e) Lactate racemization operon (*lar*) with log_2_(FC) (RM-acidic versus RM) and CRISPRi screening results for each gene.

**(i) Droplet-based CRISPRi identifies distinct, conditionally essential genes compared to batch CRISPRi.** One disadvantage of batch culture CRISPRi as performed is the inability to identify growth defects in knockdown strains that may be masked due to metabolic cross-feeding or the secretion of other public goods. To examine what new insights could be gained by circumventing this limitation, we performed a droplet-based CRISPRi under acid stress. Here, individual knockdown strains are captured in droplets containing growth medium, grown individually, and then pooled for sequencing for quantitation (Fig. 6a).

Our pilot study was based on data suggesting that bacteria can be grown in aqueous droplets focused by a fluorinated hydrocarbon oil stream (65). While we noted a technical limitation in the lower diversity of the knockdown library recovered after droplet-based growth (likely due to incomplete capture of knockdown strains in droplets), we identified a distinct set of genes vital for growth in acidic medium.

Consistent with our batch CRISPRi screening, condition-specific hits here are those that are not identified as a putative essential gene (medium or high confidence). Additionally, to help control for differences in gene fitness due to droplet growth, genes were only deemed an acid-specific droplet “hit” if they were not also identified in a droplet-based rich medium screen. Fifty genes were identified as acid-specific hits by at least two independent guides (Table S4), including genes involved in transport, energy production and conservation, or cell wall biogenesis; 14 of the 50 hits had no or poor functional annotation. Several of our identified hits in the droplet-based assay have been previously implicated in response to acid stress, including nitrate/nitrite reductases (66), multiple amino acid permeases, and a Na^+^/H^+^ transporter (reviewed in reference 64). Comparatively, 96 genes were identified as acid-specific hits by at least two independent guides in batch growth (Table S5), including ABC transporters and genes involved in coenzyme metabolism, cell wall biogenesis, energy production and conservation, or amino acid metabolism (among others), with 21 hits having no or poor functional annotation. As with our droplet-based assay hits, many of these batch hits have been previously identified in response to acid stress, including multiple amino acid permeases, arginine kinase (67), and carbamate kinase (*arcC*) (68). Surprisingly, only 17 genes were identified by both approaches, including transporters (e.g., a Na^+^/H^+^ antiporter), genes involved in cell wall biogenesis, and four genes with poor/no annotation (Table 3). Although we found the two data sets sparsely overlapping, these 17 genes are likely high confidence hits for validation.

**TABLE 3:** Genes identified by both batch and droplet-based CRISPRi

Category	Tü3298 ID^*a*^	Name	Function
Transporters	Tü3298_257	Amino acid permease family protein	Amino acid metabolism and transport
	Tü3298_1876	Amino acid permease family protein	Amino acid metabolism and transport
	Tü3298_2292	ABC transporter family protein	Inorganic ion transport: ABC-type metal ion transport system, ATPase
	Tü3298_321	ABC transporter family protein	Inorganic ion transport: ABC-type cobalamin/Fe^3+^-siderophores
	Tü3298_300	Multicomponent Na^+^:H^+^ antiporter subunit D	Inorganic ion transport and energy production
Cell wall biosynthesis	Tü3298_2288	Glycosyl transferases group 1 family protein	Cell wall biogenesis
	Tü3298_533	d-Alanyl-lipoteichoic acid biosynthesis protein DltD	Cell wall biogenesis: protein involved in d-alanine esterification
	Tü3298_253	Linear amide C-N hydrolases, choloylglycine hydrolase family protein	Cell wall biogenesis: penicillin V acylase and related amidases
Unknown function	Tü3298_403	7-Cyano-7-deazaguanine reductase	Enzyme related to GTP cyclohydrolase I
	Tü3298_1396	Radical SAM^*b*^ superfamily protein	Predicted Fe-S oxidoreductase
	Tü3298_102	Major facilitator superfamily protein	Unannotated
	Tü3298_181	kxYKxGKxW signal peptide domain protein	Unannotated
Other	Tü3298_2206	PTS^*b*^ system mannose-specific EIIBCA component	Carbohydrate metabolism: phosphotransferase system
	Tü3298_864	Ribonuclease III	Transcription
	Tü3298_246	AMP-binding enzyme family protein	Lipid metabolism: acyl-CoA synthetases (AMP-forming)/AMP-acid ligases II
	Tü3298_270	Protein IolS	Energy production and conservation: predicted oxidoreductase
	Tü3298_843	Phosphopantothenoylcysteine decarboxylase phosphopantothenate-cysteine ligase	Coenzyme metabolism

a Seventeen genes were identified as a hit by two or more guides in both the batch-based and droplet-based CRISPRi screens, indicating a high-confidence hit.

b SAM, *S*-adenosylmethionine; PTS, phosphotransferase system.

10.1128/mbio.02632-22.5 TABLE S4 Genes identified by CRISPRi under a droplet acid condition. Download Table S4, XLSX file, 0.01 MB. Copyright © 2022 Spoto et al. 2022 Spoto et al. https://creativecommons.org/licenses/by/4.0/ This content is distributed under the terms of the Creative Commons Attribution 4.0 International license.

10.1128/mbio.02632-22.6 TABLE S5 Genes identified by CRISPRi under a batch acid condition. Download Table S5, XLSX file, 0.01 MB. Copyright © 2022 Spoto et al. 2022 Spoto et al. https://creativecommons.org/licenses/by/4.0/ This content is distributed under the terms of the Creative Commons Attribution 4.0 International license.

Finally, we examined gene hits unique to a method (i.e., “droplet selective” versus “batch selective” hits). KEGG enrichment analysis identified significant enrichment of pyruvate metabolism and microbial metabolism in diverse environments in droplet-selective hits and ABC transporters in batch-selective hits (Fig. 6b). In addition to these enriched KEGG categories, we also identified individual genes that were exclusive to droplet-based or batch screening, such as the nitrate/nitrite reductase genes seen exclusively in our droplet screen. We speculate that disruptions in pyruvate metabolism lead to altered fermentative byproduct production in the confined droplet space where there is less diffusion, with the corollary that increased accumulation of metabolic byproducts may lead to a lower tolerance to existing acid stress. Similarly, we postulate that the identification of the nitrate/nitrite reduction systems exclusively in the droplet-based screen may be related to ammonium production and export under the confined droplet condition. Our basis for this hypothesis includes previous data indicating that nitrate/nitrite reductase genes are upregulated in response to acid stress in S. epidermidis and are thought to contribute to acid tolerance through the production of basic ammonia (66). In addition, some microbial species combat acid stress through secretion of this excess ammonia, which raises the pH of the immediate extracellular environment (69). Interestingly, we observed a significant clumping phenotype under this condition (Fig. S3a to c), suggesting that cells preferentially grow in close proximity (versus complete planktonic growth). We speculate that in batch CRISPRi screening, nitrate/nitrite reductase-knockdown strains may not demonstrate a fitness defect if they grow in close proximity to other strains that are producing ammonium (which would raise the localized pH or be available for import by deficient strains). This is in contrast to the homogeneity of droplets, in which cells growing in close proximity are composed of a single knockdown strain (Fig. S3a to c) and are not thereby influenced by extracellular products of different knockdown strains. Although speculative, these proposed mechanistic links could partially explain the distinct hits that we observe in the droplet-based assay (nevertheless, we note that technical differences between the two approaches also likely have some effect). Overall, our results suggest that there is a utility for both the batch- and droplet-based approaches in identifying genes of interest using CRISPRi, although future investigations should focus on improving technical limitations of the droplet system (e.g., the lower library diversity resulting from incomplete capture).

10.1128/mbio.02632-22.9 FIG S3 (a) Single S. epidermidis cells captured in droplets. Single cells can be visualized in droplets at the start of the droplet-based CRISPRi screen. Note that most droplets are empty, ensuring that most cells are captured singly within a droplet. Left, artificially colored postimaging. Right, original image for reference. (b) Multiple S. epidermidis cells growing within a droplet in TSB. After 20 h of growth, multiple S. epidermidis cells can be visualized multiplying within a droplet in the plain TSB (RM) condition. Note that individual cells can be visualized independently and do not demonstrate a clumping phenotype. Left, artificially colored postimaging. Right, original image for reference. (c) Multiple S. epidermidis cells growing within a droplet in acidic TSB. After 20 h of growth, multiple S. epidermidis cells can be visualized multiplying within a droplet under the acidic TSB (RM) condition. Note the clumped phenotype. Left, artificially colored postimaging. Right, original image for reference. Download FIG S3, TIF file, 0.6 MB. Copyright © 2022 Spoto et al. 2022 Spoto et al. https://creativecommons.org/licenses/by/4.0/ This content is distributed under the terms of the Creative Commons Attribution 4.0 International license.

**(ii) RNA-seq identifies genes enhanced and repressed in acidic medium.** We then sought to integrate gene fitness data with transcriptomic analysis in the same acidic environment. Examining first the RNA-seq data, KEGG pathway analysis identified significant enrichment of pathways involved in amino acid metabolism, carbohydrate metabolism, and environmental information processing (Fig. 6c). Specifically, significantly upregulated pathways by generally applicable gene set enrichment (GAGE) analysis included (i) arginine biosynthesis, (ii) microbial metabolism in diverse environments, (iii) biosynthesis of amino acids, (iv) 2-oxocarboxylic acid metabolism, (v) valine, leucine, and isoleucine biosynthesis, (vi) ABC transporters, (vii) histidine metabolism, (viii) tricarboxylic acid (TCA) cycle, and (ix) carbon metabolism. These results are largely consistent with previous studies in S. aureus that suggest a role for amino acid metabolism (particularly arginine), transport, and TCA cycle alterations in acid tolerance (reviewed in reference 70). Among the mostly highly upregulated genes (log_2_[FC] > 2), we identified efflux pumps, a H^+^/Na^+^ antiporter, and components of the arginine deiminase system, which have a demonstrated role in responding to acid stress (71). Surprisingly, however, four genes of the *potRABCD* operon, which encodes a spermidine/putrescene uptake system, were highly upregulated (log_2_[FC] *potR *= 2.0, *potB *= 2.1, *potC *= 2.08, and *potA = *1.60). *speG*, which encodes a spermidine acetyltransferase, and *speC*, which encodes an ornithine decarboxylase, were also modestly upregulated under acid stress (log_2_[FC] *speG*= 0.557 and *speC *= 0.343). While spermidine and putrescene protect against stress in E. coli via their role in the glutamate decarboxylase acid response system (72), this system does not appear to be present in our strain of S. epidermidis.

**(iii) Leveraging both CRISPRi and RNA-seq analysis identifies a subset of genes enriched and essential in response to acid stress.** Previous reports have shown that condition-specific essential genes are not necessarily overexpressed under that condition, possibly to protect vital genes from large fluctuations in transcription (73, 74). However, by identifying subsets of genes whose expression is elevated and that are essential for growth under a stressor, we can begin to conceptually link how a strain’s transcriptomic response is related to survival under stress. Under acid stress, we identified 35 genes both enriched and essential under acid stress (Fig. 6d). Many of these genes are involved in transport (10 of 35) or cell wall biosynthesis (5 of 35). Of particular interest was the *lar* operon (Fig. 6e), which encodes a nickel-dependent lactate racemase, and several genes involved in the processing and maturation of the enzyme. The role of this lactate racemase is not fully elucidated beyond its role in lactate-consuming microbes. For example, evidence in Lactobacillus plantarum demonstrated that lactate racemase plays a role in cell wall biosynthesis through its generation of d-lactate from l-lactate, consequently conferring resistance to high levels of vancomycin. As previously discussed, modification of the cell wall has been shown to play a role in response to acid stress in E. coli (75). Taken together, we speculate that the importance of the lactate racemase operon under acid stress in S. epidermidis may similarly be related to cell wall structure.

## DISCUSSION

Until the advent of CRISPRi, large-scale studies of gene function have been largely limited to genetically tractable model organisms. Circumventing direct gene editing, highly customizable to a genome of interest, and easily scalable, CRISPRi is well suited to study microorganisms such as S. epidermidis, which, despite its ubiquity and unquestionable human heath relevance, has not seen as deep a probe of its function as needed to begin to understand its lifestyle versatility. Here, we present a high-throughput demonstration of CRISPRi in a ubiquitous human microbe, complemented with transcriptomics across a diverse array of conditions. Additionally, we piloted an application of high-throughput droplet-based CRISPRi to study gene function in any microbe. Our findings provide a reference both for other staphylococcal researchers and the prokaryotic community more generally with our benchmarking analysis for large-scale CRISPRi, which suggests that the behavior of CRISPRi screens are likely species and/or strain specific (Text S1 and Fig. S1 in the supplemental material).

As both a colonizer and pathogen that thrives across human skin and multiple infection niches, S. epidermidis adapts to a wide range of environmental stressors. Our fitness screen across multiple conditions in S. epidermidis allowed us to identify putative essential genes, multistress response genes, and condition-specific essentials. These data then allowed us to make numerous new hypotheses into the biology of S. epidermidis.

We made a first investigation into the lifestyle versatility at the gene fitness level to identify genes crucial to survival in a diversity of environmental conditions, a prequel to subsequent studies expanding to infection and colonization environments. In contrast to traditional gene essentiality screens conducted in rich medium, we leveraged screen data across diverse environmental conditions and identified genes and pathways, such as amino acid metabolism, that were more broadly vital for growth and adaptability to multiple environmental pressures. For example, we identified staphylopine biosynthesis and dependent metal transport as crucial stress-response functions. Future investigations might focus on the role of staphylopine in colonization and infection niches of S. epidermidis; is staphylopine dispensable for colonization of healthy skin but crucial to combat infection-associated stress? Similar investigations could focus on any one of these 26 stress-responsive genes, particularly those with no/poorly annotated function, to determine their importance in healthy colonization versus infection.

Additionally, we complemented our CRISPRi screen with RNA-seq to investigate lifestyle flexibility by studying both fitness and transcriptional plasticity. Interestingly, we identified upregulation of the quorum sensing *agr* locus and three PSMβ genes under the rich anaerobic growth condition, suggesting a role for these virulence determinants in a hypoxic infection niche. Additionally, we observed a surprising enrichment of bacteriocin synthesis/export and nitrate/nitrite reductase genes across three hyperosmotic stress environments, a proxy for the hyperosmotic conditions of sweat. We postulate that nitrate/nitrite reductase upregulation is an adaptation to use nitrogen from urea-rich sweat as an energy source, highlighting the role of transcriptional plasticity and an example of the decoupling of gene essentiality and environmental adaptation, as these genes were not crucial for its survival in this environment. This interplay between nitrogen metabolism and hyperosmotic stress adaptation is another potential link for follow-up mechanistic studies.

S. epidermidis must combat various pH levels on skin, which generally range from ~4.1 to 5.8 but are affected by skin site, individual, and lifestyle habits (62, 63). Thus, acid stress adaptation is an important component of S. epidermidis colonization and lifestyle flexibility on human skin. Our results led us to some intriguing hypotheses into the response to acid stress in S. epidermidis. First, polyamine accumulation occurs via a GDAR (glutamate decarboxylase-dependent acid resistance) independent pathway. Polyamines are known for their role in the stress response (particularly oxidative stress) across multiple organisms, but the literature regarding their function in acid stress tolerance is sparse. Chattopadhyay and Tabor et al. demonstrated that polyamines protect against acid stress in E. coli (72) but proposed a mechanism via the GADR system, which has no homolog in our strain of S. epidermidis. Second, ammonium production by nitrate/nitrite occurs to raise the localized pH, which we only identified by droplet-based CRISPR. Third, cell wall synthesis and modification occurs, especially the lactate racemase (*lar*) operon, which has previously been demonstrated as important in acid tolerance in E. coli (75).

As with any knockdown screening method, inherent technical considerations of CRISPRi resulting in incomplete or ineffective knockdown limit our ability to draw fully conclusive results. First, we acknowledge that we did not target all predicted coding sequences. To the best of our efforts, which involved greater than 100 transformations into E. coli and greater than 100 subsequent transformations into the intermediary S. aureus, we found diminishing returns in efforts invested in guide recovery, as guides are lost, presumably at random, at each step. In addition, 84 coding sequences do not have any NGG (N is any nucleotide base. G is guanine) targets. Future efforts could supplement pools with subpools of synthesized guides to improve genome coverage or use PAM (protospacer adjacent motif)-less dCas proteins to improve guide coverage across the genome (76, 77).

Second, inherent technical considerations may limit the ability of the screen to fully identify essential genes, including transcript stability, transcript and protein half-life, and incomplete knockdown. Additionally, the organization of prokaryotic genes in operons and thus polarity effects cannot be overlooked as a biological consideration. As genome annotation of S. epidermidis improves, we anticipate that our group and others will be able to revisit the data presented here for improved validation of this phenomenon and others. For the technical and biological reasons presented here, validation of high-quality hits through rigorous individual testing is still required before definitive conclusions can be drawn. Additionally, while our droplet-based CRISPRi pilot study demonstrated the value in this complementary approach, we acknowledge that there may be potential differences between growth in droplet and batch culture. For instance, while droplets contained by fluorinated hydrocarbons are able to diffuse oxygen (78), and we confirmed that this is sufficient to support the growth of S. epidermidis, other data suggest that oxygen concentration within droplets fluctuates and may not support the growth of more fastidious bacteria that require stable oxygen concentrations (78). Overall, rather than serving as a conclusive study on gene function, we deem our CRISPRi data here to be a high-throughput screening resource; compared to individual knockout studies, its major advantage is its high-throughput format and the ability to quickly scale up to screen in multiple conditions, as performed.

Third, we note that a major technical limitation to the CRISPRi approach may be low transformability of this or other species. Future work should focus on increasing transformation efficiency or otherwise maneuvering around this roadblock. Indeed, there have been several advances in this field already that may be well applied for large-scale CRISPR-Cas targeting in otherwise intractable species (79, 80).

In summary, our complementary approaches investigating gene fitness and gene plasticity across a diverse set of physiologically relevant conditions allowed us to make new biological insights into a keystone skin microbe. We also provided a real-world demonstration of both the promise and pitfalls of CRISPRi for high-throughput fitness profiling. Future studies building on the accessibility and scalability of these approaches will further mechanistic investigation into the biodiversity of S. epidermidis, including the functional consequences of its extraordinary genetic diversity, as well as investigation into the tremendous biodiversity of its greater microbial neighbors.

## MATERIALS AND METHODS

### CRISPRi pooled library creation.

**(i) Vector design.** Our CRISPR-dCas9 vector, which includes all components required for CRISPRi, has been previously validated (34). The CRISPR-dCas9 vector is comprised of the dCas9 sequence (derived from pDB114dCas9 [81]) under the control of an anhydrotetracycline (aTc)-inducible promoter (derived from pRAB11 [82]), a custom-designed dCas9 handle composed of a CRISPR RNA and a transactivating small RNA fusion, two BsaI sites for ligation of single guide RNA (sgRNA) guides, and a chloramphenicol (Cm) resistance maker for selection.

**(ii) sgRNA design.** sgRNAs 20 nucleotides in length were designed to target every NGG PAM site on both strands of the S. epidermidis Tü3298 genome using a customized version of the GuideFinder R scripts.

The Tü3298 strain of S. epidermidis was chosen for our screen for both technical and biological reasons. First, it represents an interesting skin commensal strain due to its production of the lantibiotic epidermin (83). Second, and importantly, the strain is amenable to the phagemid plasmid transfer protocol (36). Guides with GC content of <30% were discarded, but no other filtering was performed after guide design. The identified sgRNA sequences were flanked with custom sequences containing BsaI restriction enzyme sites (for generation of single-stranded ends) and primer sites for PCR amplification (see Data availability for access to vector design). In addition to 60,157 targeting guides, 100 scramble guides, were ordered from Twist Biosciences low-mass oligonucleotide pool. The 100 scramble guides were designed by subjecting randomly generated sequences to a BLAST search against the S. epidermidis Tü3298 genome. Only guides that did not match anywhere in the genome were selected as a scrambled, nontargeting guide. All guides containing flanking sequences as ordered are available as described in Data availability.

**(iii) Pooled library construction.** The pool was amplified with custom PCR primers (forward: 5′-GCTGTTTTGAATGGTCCC-3′; reverse: 5′-CCGTTATCAACTTGAAAAAGTGG-3′) using New England BioLabs (NEB) Q5 high-fidelity DNA polymerase, according to manufacturer’s instructions, with an annealing temperature of 58°C and an extension time of 30 s for 10 cycles (Twist Biosciences recommends cycling between 6 and 12 times). The amplified pool was visualized on agarose gels, digested with BsaI to produce single-stranded overhangs, and ligated into a BsaI-digested pidCas9 targeting vector containing complementary single-stranded overhangs. To enhance transformation of our ligation library, ligation reactions (using NEB T4 ligase) were performed at 16°C overnight in bulk and mixed in 0.5× volume with AMPure beads to remove impurities and concentrate the ligation 20× in nuclease-free water. The cleaned and concentrated ligation library was transformed into NEB 5α electrocompetent E. coli, and transformants were collected directly from the plate by flooding the plate with TSB/Cm. We estimated that 250,000 colonies were recovered. The Qiagen miniprep kit was used for plasmid purification from the collected colonies according to manufacturer’s instructions, except that several lysate reactions were loaded on to one column sequentially before elution in nuclease-free water. The isolated plasmids were concentrated to >500 ng/μL before transformation into electrocompetent S. aureus PS187 to ensure a high number of transformants. S. aureus PS187 transformants were collected by plate scraping and used directly for phagemid transfer. We estimated that 115,000 colonies were recovered across approximately 100 plates. Plasmids were transferred to S. epidermidis Tü3298 from S. aureus PS187 via phagemid transduction as described elsewhere (36). Briefly, bacteriophage Φ187 lysate and the plasmid-bearing S. aureus cells were incubated together for 30 min at 37°C without agitation and then moved to 30°C with gentle agitation until the mixture cleared, indicating bacterial lysis by the phage, after approximately 3 h. The phage lysate was centrifuged to pellet debris and was filter sterilized twice with 0.22-μm filters before storage at 4°C until use. The phage lysate containing plasmid-bearing phage particles was incubated with S. epidermidis Tü3298 cells for 30 min and plated on tryptic soy agar (TSA)/chloramphenicol plates for selection of transformants. We estimated that 150,000 colonies were recovered. Transformants were collected by the plate scraping method, diluted to an optical density (OD) of ~10 in TSB/Cm, and saved as glycerol stocks at −80°C.

### CRISPRi screening.

**(i) Culture conditions.** Our CRISPRi screening was conducted under 24 diverse stress and nutrient-limiting conditions. The base medium conditions are described as follows. For alternations to these base medium conditions, including addition of stressors (e.g., hydrogen peroxide), see Table 1.

Defined medium (DM) was composed of Iscove’s modified Dulbecco’s medium (IMDM) with the following additions: 20 μg/mL Cm (for plasmid selection), 5g/L glucose, 20 mg/L adenine sulfate, 20 mg/L guanine HCl, 1:100 of minimal essential medium (MEM) 100× nonessential amino acids solution, 1:50 MEM 50× essential amino acids solution, and 25 μM FeCl_3_ (unless otherwise indicated, as in low- and high-iron conditions. pH was adjusted to 7.0 with 1 N NaOH.

Minimal medium was composed of M9 minimal medium, according to the Cold Spring Harbor protocol (84), with the addition of FeCl_3_ to 25 μM, 20 μg/mL Cm (for plasmid selection), 2 mg/L thiamine HCl, and 2 mg/L nicotinic acid. This solution was adjusted with various concentrations of glucose (1% or 0.1% weight/volume [wt/vol]) and amino acids (OmniPure Casamino Acids [CAA], 0.01% or 1% [wt/vol]).

**(ii) CRISPRi growth assays.** For each condition, medium containing 0.1 μM aTC (for induction) and 20 μg/mL chloramphenicol (for plasmid selection) was inoculated 1:1,000 with glycerol stock of the knockdown pool. The cultures were grown aerobically with a flask:medium ratio of 5:1 at 37°C with shaking, unless otherwise indicated. A list of tested conditions is available in Table 1. Two aliquots were taken at separate time points targeting early- and early-mid-exponential phase for each condition. Before each assay,16- to 36-h growth curves were determined for each condition using the Biotek Cytation imaging multimode reader to estimate growth kinetics. After sampling, cells were centrifuged, the supernatant was removed, and the pellets were frozen at −20°C until preparation for sequencing.

**(iii) Small-scale multiplexed CRISPRi.** The 4-strain pool was made by mixing equal volumes of glycerol stock of previously created individual knockdown strains. For the 500-knockdown-strain pool, guides were designed to gene body regions using a customized version of our GuideFinder script. Guides were ligated into our knockdown vector using the same approach for individual strain creation, as we previously described (34). Briefly, guides were ordered as two single-stranded oligonucleotides (forward and reverse strands). Oligonucleotides were phosphorylated, annealed, and ligated into our BsaI-digested pidCas9 targeting vector. The procedure for transformation into E. coli and S. aureus and phagemid transfer into S. epidermidis is the same as described for the large-scale pools. Culture conditions for both small knockdown pools are the same as described for the large pool analyses.

**(iv) CRISPRi sequencing.** For each condition, frozen cell pellets were washed in 1× phosphate-buffered saline (PBS), subjected to boiling alkaline lysis in 100 mM NaOH at 95°C for 5 min, and neutralized with 1 M Tris (pH 8.0) before PCR amplification of the crude lysate. Custom PCR primers were designed to work with the Illumina sequencing system to amplify the sgRNA region of the vector via primer binding sites on the pidCas9 vector upstream and downstream of the guide region (data are provided as described in Data availability, including the primer design scheme and full list of primers used for sequencing). In brief, custom primers contain an adapter sequence, a Nextera index sequence for unique dual indexing (UDI), a sequencing primer, a stagger to maintain sequence diversity, and a PCR primer sequence (targeting upstream or downstream of the sgRNA guide sequence) for amplification of the product before sequencing. PCR was performed with NEB Q5 high-fidelity DNA polymerase, according to manufacturer’s instructions, with an annealing temperature of 58°C and an extension time of 30 s for 25 cycles. The PCR product was visualized by agarose gel electrophoresis, and the concentration was assessed with Qubit fluorometric quantitation using a negative PCR as a baseline and was adjusted to 4 nM with nuclease-free water. Samples were pooled and cleaned with AMPure beads (sequential 1.6× and 0.9× bead concentration cleaning steps) before sequencing. Sequencing was performed on the Illumina HiSeq and NovaSeq platforms targeting a read depth of ~10 to 20 million reads/sample.

**(v) Guide hit identification.** Reads were trimmed to the sgRNA sequence using cutadapt (v1.18) (85) and aligned to a database containing all possible guides with BLASTn (NCBI blast+ v2.6.0) (86) against a database of all designed sgRNA sequences (the read was considered a match with 100% identity and 100% coverage). Based on our design (described above), each guide was placed into one of the following categories based on their targeting location: (i) targeting a gene on the nontemplate strand, (ii) targeting a gene on the template strand, (iii) targeting a nonprotein-coding sequence, or (iv) targeting multiple genomic locations. We expected that guides targeting genes on the nontemplate strand will lead to more profound gene knockdown (although differences in guide efficacy affect extent) than guides targeting the template strand. We assumed guides targeting the template strand will not lead to fitness defects of vital genes. We assumed guides targeting the nontemplate strand of vital genes will often lead to fitness defects and acknowledge that not every guide will be effective in knocking down a gene. The raw read counts were input into a custom R script, and we used DESeq2 (v1.26.0) (87) to calculate a log_2_(FC) for each guide; a default median of ratios normalization method was used. The adaptive shrinkage estimator was used for log_2_(FC) shrinkage via method=ashr (88). The log_2_(FC) reflects the difference in the relative abundance of the guide from each sampled time point to the initial pool. This analysis was performed to identify hits for each condition unless otherwise indicated (e.g., comparing induced versus uninduced samples for rich medium pool characterization). To determine guides that were significantly reduced in the sampled time points compared to in the initial pool (“guide hits”), an FDR cutoff for each specific condition was determined (FDR that corresponds to the top 25% smallest FDR values). To be considered a “guide hit,” a guide must meet this FDR threshold and have a log_2_(FC) of ≤1. Based on literature and our own experience, which indicates that not every guide targeted to a vital gene on the nontemplate strand will result in a fitness defect, we did not require that every guide (or any percentage of total guides) targeting a particular gene on the nontemplate strand be identified as a “guide hit” in order for the gene to be considered vital for growth. Along the same lines, we opted not to use median log_2_(FC) of guides targeted to a gene to determine gene fitness.

**(vi) Investigation of essential and nonessential genes in rich medium.** To investigate the utility of our CRISPRi knockdown pool, we screened our pool in plain rich medium (TSB) and plotted the distribution of log_2_(FC) values for guides targeting essential and nonessential genes. Our set of validating essential and nonessential genes was determined by homology (>30% homology over >90% sequence length) to known essential and nonessential genes in S. aureus (84). Each S. aureus gene with more than one hit in S. epidermidis was interrogated independently, and the best hit was selected manually. Although there are likely some differences in essentiality between S. aureus and S. epidermidis, we believed there to be enough consistency in essentiality between the species to indicate whether our screen was feasible in this preliminary investigation.

**(vii) Essential gene hit identification.** Essential gene hits were binned according to high, medium, and low confidence as follows. High-confidence genes were those that were identified by at least two “guide hits” in greater than two-thirds of all conditions. Medium-confidence genes were those that were either (i) identified by at least two “guide hits” in one-half of all conditions or (ii) were identified by one “guide hit” in two-thirds of all conditions. Low-confidence genes were those that were identified by at least one “guide hit” in one-half of all conditions. Gene accumulation curves for high-, medium-, and low-confidence essential genes across conditions were generated using the vegan R package (v.2.5.7) (89). One guide from each of these high-confidence gene hits was randomly selected for individual knockdown strain creation and was created and individually assayed as described above in CRISPRi growth assays.

**(viii) Conditional gene hit identification.** Owing to the different growth rate of S. epidermidis across conditions and the level of growth that each of these conditions supports, we determined that it was not appropriate to compare absolute log_2_(FC) values across conditions (i.e., we do not suggest that a gene has lower fitness in one condition versus another based on the degree of log_2_[FC] of the guides). To determine condition-specific gene hits, we identified hits for each condition (described above) and removed any putative essential genes that we identified with medium or high confidence. Thus, “condition-specific” gene hits are not those that are exclusively identified in one condition but are those that are not generally identified across conditions (i.e., are not a putative essential gene).

**(ix) Multistress response gene identification.** To find multistress response genes, we searched for hits across multiple stress conditions in rich medium. It is likely that nutrient availability affects gene fitness under stress, and so we restricted our analysis to stress screens performed in rich medium to reduce the effect of background medium condition within our rich medium stress conditions. These included the subinhibitory antibiotic (RM-mupirocin, RM-ciprofloxacin, and RM-vancomycin), hyperosmotic (RM-sucrose, RM-glycerol, RM-urea, and RM-salt), pH (RM-acidic and RM-alkaline), heat (RM-42), sodium hypochlorite (RM-HOCl), and hydrogen peroxide (RM-H_2_O_2_) stress conditions. A gene was considered a “multistress response” gene if it was (i) identified as a hit (see above; briefly, by two or more independent guides with a log_2_[FC] of <−1) in 6 or more of these 13 stress conditions, (ii) not identified as a hit in plain rich medium (TSB), and (iii) not identified as a putative essential gene (medium- or high-confidence bin).

### Droplet-based CRISPRi.

** (i) Droplet generation and growth assay.** We piloted a droplet-based CRISPRi assay in plain rich medium (TSB) and acidic rich medium (RM-acidic; pH 4.8). The RainDrop Droplet Digital PCR system by RainDance Technologies was used to generate droplets containing the growth medium with single S. epidermidis cells bearing the CRISPR-dCas9 vector. Droplets were generated using the standard droplet generation oil from Bio-Rad (fluorinated hydrocarbon). We calculated the number of S. epidermidis cells necessary to generate primarily empty droplets to avoid capture of multiple cells (~2 × 10^4^ cells/μL), and we confirmed the presence of single-cell droplets by light microscopy (Fig. S3a). After droplet capture, the cultures were incubated at 37°C, and aliquots were taken periodically to assess growth of the cells in the droplets. Based on our batch culture results, we anticipated that growth in RM-acidic would be much slower than growth in plain rich medium. Rich medium samples were grown for 8 h, and RM-acidic samples were grown for 20 h; cultures were observed throughout via sampling and light microscopy to determine appropriate timing. Light microscopy images (Fig. S3b and c) demonstrated bacterial growth in the droplets. At the end of each assay, droplets were broken by the addition of 1*H*,1*H*,2*H*,2*H*-perfluoro-1-octanol. Amplicons were generated and sequenced as described above in CRISPRi sequencing, with the following modification: after droplet lysis, the oil phase was removed and centrifuged to pellet cells. Pellets were frozen at −80°C until use.

** (ii) Acid stress-specific gene hits identification.** Acid stress-specific “guide hits” were determined as described above for our batch CRISPRi screen. Genes under this condition were identified as a hit if they met all three of the following conditions: (i) the gene was identified as a hit by two or more guides, (ii) the gene was not identified as a putative essential gene (by medium or high confidence), and (iii) the gene was not identified as a hit by two or more guides in the rich medium droplet condition. We included this last constraint in our droplet-based CRISPRi screen to control for potential differences in gene fitness in droplet versus batch rich medium.

### Transcriptomic analysis.

**(i) Culture conditions.** For each condition, medium containing 0.1 μM aTC was inoculated 1:1,000 with glycerol stock of the empty (nontargeting) vector. When possible, the same batch of medium was used for the genome-wide screen assay, and the RNA-seq assay and the samples were grown concurrently. The cultures were grown aerobically with a flask:medium ratio of 5:1 at 37°C with shaking, unless otherwise indicated. Two aliquots were taken at separate time points for each condition targeting early- and mid-exponential phase. When necessary, cultures were moved into smaller flasks after sampling to maintain the 5:1 flask:medium ratio. Cells were centrifuged, washed in 1× PBS, resuspended in TRIzol, and frozen at −80°C until RNA extraction.

**(ii) RNA extraction, library preparation, and sequencing.** RNA samples were extracted using the Qiagen RNeasy Plus minikit according to manufacturer’s instructions with the following modifications. Samples were thawed on ice, centrifuged to pellet and to remove TRIzol supernatant, and resuspended in RLT Plus buffer with 0.1-mm glass beads (autoclaved twice before use). Samples were bead-beaten using the Qiagen TissueLyser for 3 min and centrifuged to pellet beads. The supernatant was mixed with an equal volume of 70% ethanol. This mixture was applied to the Qiagen RNeasy column and washed with appropriate buffers according to manufacturer’s instructions. An on-column DNase digestion was performed according to the Qiagen RNeasy protocol using Qiagen RNase-free DNase. Samples were eluted in nuclease-free water according to manufacturer’s instructions. RNA was frozen at −80°C until sequencing preparation. All RNA isolation steps were performed in a sterile hood following the protocol to reduce RNA degradation (i.e., use of disposable plasticware/glassware, 2× autoclaved glass beads, and RNaseZap RNase decontamination solution use on all surfaces). RNA quality was analyzed using an Agilent TapeStation and quantified by Qubit according to manufacturer’s instructions. RNA integrity number (RIN) values ranged from 6.0 to 8.5 with a mean and median of 7.7. RNA was prepared for sequencing using the NEBNext rRNA depletion kit (Bacteria) and NEBNext Ultra II directional RNA library prep kit for Illumina according to kit instructions and sequenced on the Illumina NovaSeq, targeting 40 to 60 million reads/sample. Sequences from each sample were trimmed of adapters using Trimmomatic (v0.32) (90), and rRNA, tRNA, phiX sequences, and host contaminants were filtered out using BWA-MEM (v0.7.7) (91). BWA-MEM (v0.7.7) was used to align clean reads to the reference Tü3298 genome. Mapped reads were assigned using featureCounts (v1.6.0) to generate a count table of reads to coding sequences (92).

**(iii) Differentially expressed gene identification.** DESeq2 was used to identify differentially expressed genes based on our input count tables. Unless otherwise indicated, plain rich medium (TSB) was set as the reference for any stress conditions conducted in rich medium. The plain defined medium condition was set as the reference for any stress screen under this condition. M9 minimal medium supplemented with 1% glucose and 1% Casamino Acids (CAA) was set at the reference for any screen conducted in M9 minimal medium (concentrations of glucose and/or Casamino Acid additions vary). The log_2_(FC) values were calculated with the standard median of ratios normalization method; the adaptive shrinkage estimator was used for log_2_(FC) shrinkage via method=ashr (88). Principle-coordinate analysis (PCoA) plotting of normalized read counts was used as a quality control (QC) check to determine the relationship of the samples to one another. During this QC process, we found that the “mid-exponential” time points (all 3 biological replicates) for the plain rich medium (TSB) condition clustered away from the remaining samples. Based on our analysis, we believe that these rich medium samples are closer to the stationary phase and determined that it would not be appropriate to compare mid-exponential-phase time points for the remaining samples. As such, we determined that it would be more appropriate to focus our remaining analysis on just the early exponential time point for all of our samples. Using the shrunken log_2_(FC) values obtained from DESeq2, differentially enriched and repressed KEGG pathways were inferred using the GAGE package (v2.36.0). Pathview (v1.26.0) was used to generate visualizations of significantly enriched and repressed pathways as determined by GAGE.

### Data availability.

Data including (i) all raw count data and log_2_(FC) with FDR-adjusted *P* values for every guide by CRISPRi screening, (ii) condition-specific hits and putative essential genes identified by CRISPRi screening, (iii) raw count data and log_2_(FC) with FDR-adjusted *P* values for RNA-seq, (iv) custom R scripts for guide design and custom scripts for analysis (available upon request), (v) pidCas9 vector design, (vi) Tü3298 annotation, and (vii) PCR and sequencing primers are available at https://github.com/ohlab/S.epi-CRISPRi-and-RNA-Seq. All raw and processed sequencing data generated in this study are available under BioProject Accession: PRJNA900578.
