# Supplementary figures and images for: Large-Scale CRISPRi and Transcriptomics of Staphylococcus epidermidis Identify Genetic Factors Implicated in Lifestyle Versatility
Source: mBio. 2022 Nov 21;13(6):e02632-22. doi: 10.1128/mbio.02632-22 (PMC9765180; doi:10.1128/mbio.02632-22)

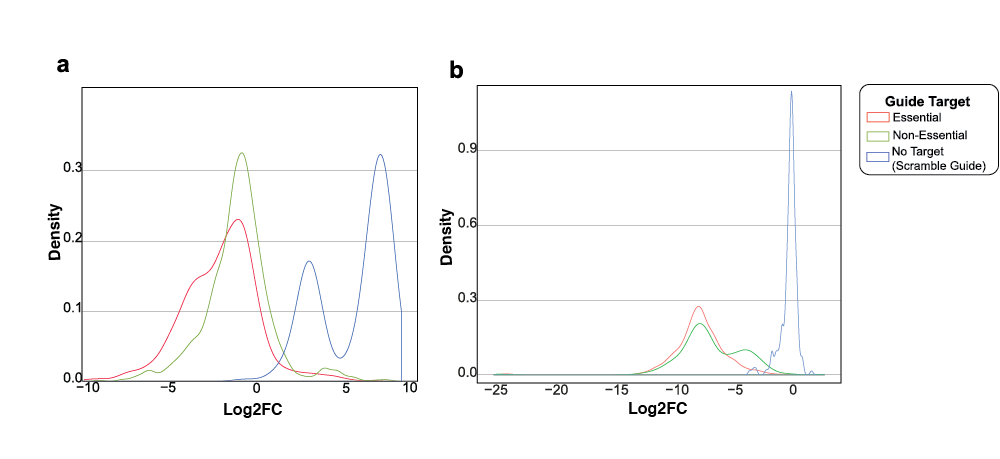

Supplement: FIG S1 [file mbio.02632-22-s0001.tif]

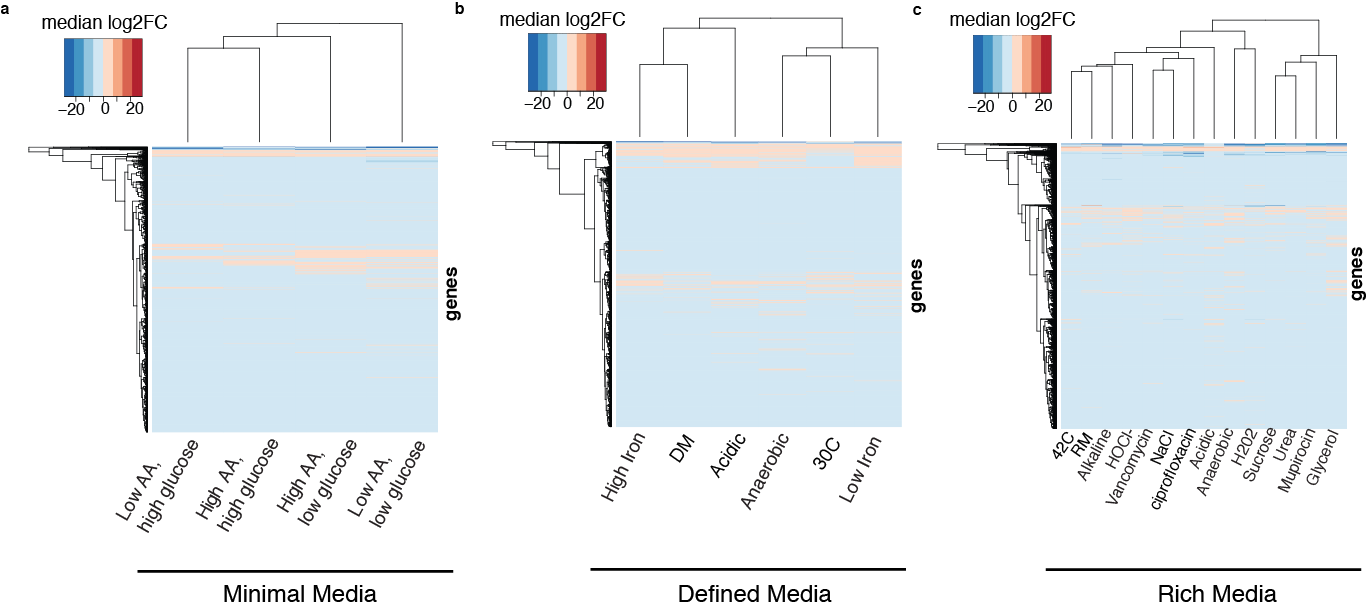

Supplement: FIG S2 [file mbio.02632-22-s0002.tif]

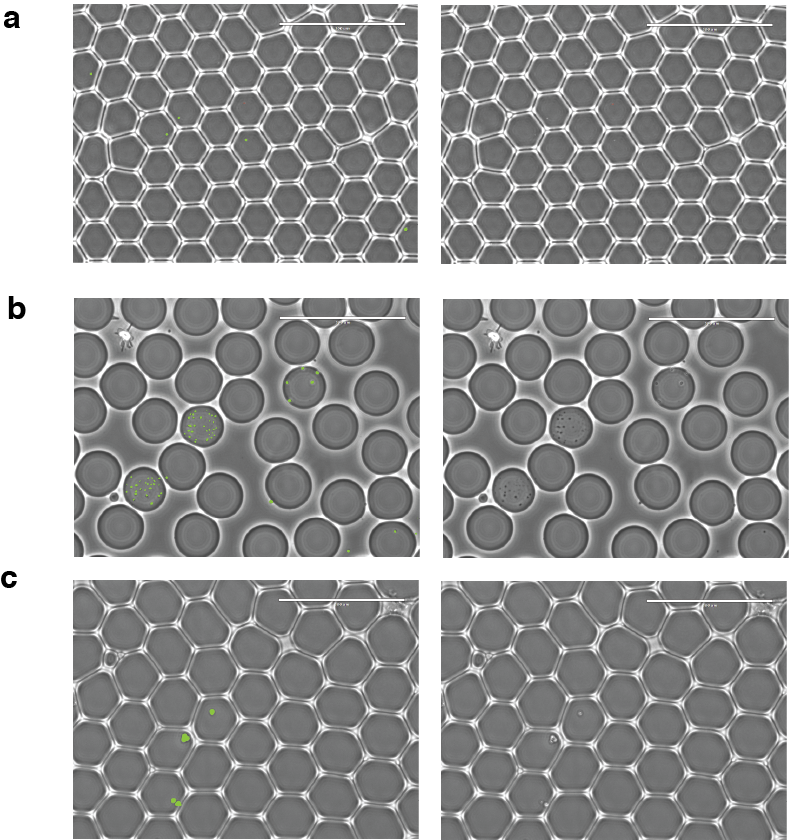

Supplement: FIG S3 [file mbio.02632-22-s0003.tif]
